# Nonidealities in CO_2_ Electroreduction Mechanisms Revealed by Automation-Assisted Kinetic Analysis

**DOI:** 10.1021/acscentsci.3c01295

**Published:** 2024-06-28

**Authors:** Joy S. Zeng, Vineet Padia, Grace Y. Chen, Joseph H. Maalouf, Aditya M. Limaye, Alexander H. Liu, Michael A. Yusov, Ian W. Hunter, Karthish Manthiram

**Affiliations:** † Department of Chemical Engineering, Massachusetts Institute of Technology, 77 Massachusetts Avenue, Cambridge, Massachusetts 02139, United States; ‡ Department of Mechanical Engineering, Massachusetts Institute of Technology, 77 Massachusetts Avenue, Cambridge, Massachusetts 02139, United States; § Division of Chemistry and Chemical Engineering, California Institute of Technology, Pasadena, California 91125, United States

## Abstract

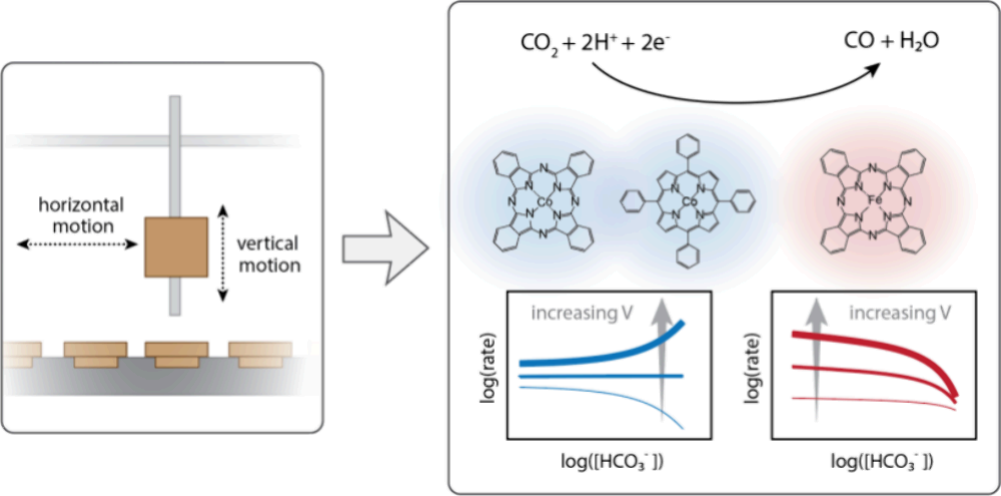

In electrocatalysis, mechanistic analysis of reaction rate data often relies on the linearization of relatively simple rate equations; this is the basis for typical Tafel and reactant order dependence analyses. However, for more complex reaction phenomena, such as surface coverage effects or mixed control, these common linearization strategies will yield incomplete or uninterpretable results. Cohesive kinetic analysis, which is often used in thermocatalysis and involves quantitative model fitting for data collected over a wide range of reaction conditions, requires more data but also provides a more robust strategy for interrogating reaction mechanisms. In this work, we report a robotic system that improves the experimental workflow for collecting electrochemical rate data by automating sequential testing of up to 10 electrochemical cells, where each cell can have a different electrode, electrolyte, gas-phase reactant composition, and applied voltage. We used this system to investigate the mechanism of carbon dioxide electroreduction to carbon monoxide at several immobilized metal tetrapyrroles. Specifically, at cobalt phthalocyanine (CoPc), cobalt tetraphenylporphyrin (CoTPP), and iron phthalocyanine (FePc), we see signatures of complex reaction mechanisms, where observed bicarbonate and CO_2_ order dependences change with applied potential. We illustrate how phenomena such as electrolyte poisoning and potential-dependent degrees of rate control can explain the observed kinetic behaviors. Our mechanistic analysis suggests that CoPc and CoTPP share a similar reaction mechanism, akin to one previously proposed, whereas the mechanism for FePc likely involves a species later in the catalytic cycle as the most abundant reactive intermediate. Our study illustrates that complex reaction mechanisms that are not amenable to common Tafel and order dependence analyses may be quite prevalent across this class of immobilized metal tetrapyrrole electrocatalysts.

## Abstract 2

Reaction rate data collected with an automated robot show that several well-studied, heterogeneous CO_2_ electroreduction catalysts exhibit significant voltage-dependent changes in reaction mechanisms.

## 1 Introduction

Reaction kinetics analysis in electrocatalysis has traditionally relied on interpretations of linearized rate data. In these analyses, one assumes that, within the rate equation, dependences on different experimental handles (e.g., voltage and concentration) can be factored separately and linearized on log–log or semilog graphs. This is the basis for typical Tafel and reactant order dependence analyses. Although linearization can provide valuable mechanistic insight with relatively light experimental data collection, it requires highly simplifying assumptions. Coverage effects, mixed rate control, and side reactions can all lead to rate behavior that is difficult to analyze using linearization.^1−4^ Given the substantial effect of voltage on reaction energy landscapes, as well as the general complexity of electrified interphases, it is reasonable to expect that many electrocatalytic reactions follow complex, nonlinearizable reaction mechanisms. For example, since many elementary charge transfer steps display an order of magnitude change in equilibrium constant with only 59 mV of voltage difference (i.e., 59 mV/dec scaling), surface coverages of reaction intermediates could drastically change even within a potential window of just a few hundred millivolts. Additionally, effects from solvent or electrolyte displacement, which are general to solid–liquid interfaces^5^ but may also have additional potential dependences,^6−8^ can further complicate reaction rate data.

On the other hand, cohesive kinetic analyses allow for consideration of the aforementioned complexities. Such analyses interrogate possible correlations between variables by using large volumes of data collected over wide ranges of reaction conditions. These data can be fit to general and complex rate equations using quantitative statistical methods.^9^ Although cohesive kinetic analyses are more common in heterogeneous thermocatalysis,^10−13^ in electrocatalysis, these analyses, as well as other analyses beyond linearization, have already helped to highlight some of the complex kinetic phenomena^14^ that underpin the CO_2_ reduction reaction (CO_2_RR). For example, a meta-analysis of reported Tafel data across many classes of CO_2_RR catalysts showed that Tafel slopes do not show a strong preference for commonly interpretable values (e.g., 120, 60, and 40 mV/dec).^15^ This pointed to the likely prevalence of additional mechanistic phenomena that are not captured with a traditional Tafel analysis. Additionally, cohesive kinetic analysis of complex reaction rate data provided evidence of mechanistic complexities such as competitive electrolyte adsorption and mixed control for CO_2_RR to CO on cobalt phthalocyanine.^16^ Finally, kinetic modeling coupled with continuum transport modeling illustrated the importance of mass transport and competing reactions for explaining complex kinetic behavior of CO_2_RR to CO at Ag.^17^ Thus, the wider use of cohesive kinetic analysis in electrocatalysis could shed light on complex kinetic behavior that has been unexplained or even missed by typical linearization-based kinetic analyses.

One barrier for implementing cohesive kinetic analysis in electrocatalysis is its heavy data requirement. Collecting the data manually can entail months of intensive yet tedious experimentation, and the data can be subject to human error or variability. On the other hand, automation can provide a tool for accelerating and standardizing such workflows. However, to date, most strategies for automation in electrocatalysis are tailored toward high-throughput catalyst or condition screening.^18,19^ For example, one automation strategy involves miniaturization, where small (order microliter) electrolyte volumes are employed for rapid materials screening within scanning droplet cells.^20−22^ However, with such small working volumes, it can be difficult to quantify reaction products; thus, these rapid screening techniques cannot be used to automate the kinetic analysis of electrocatalytic reactions that do not have 100% Faradaic efficiency. Parallelization is another strategy, where arrays of different metal compositions or electrolyte conditions are tested simultaneously.^23−25^ Parallel setups often do allow for product quantification, but quantification is typically performed either by aggregating effluents from multiple reactors or by manual workup after electrolysis. An automated kinetic analysis workflow would ideally involve online product quantification of individual electrochemical cells, which is possible but likely to be expensive in a parallel configuration. Thus, a strategy involving sequential electrochemical testing and online product quantification of well-controlled reaction conditions within geometrically well-defined reactors would be ideal for automating kinetic analysis. This automation strategy has been used to screen catalysts for the (photo)electrochemical CO_2_ reduction reaction.^26−29^ We demonstrate that a similar automation concept can be tailored to automate electrochemical kinetic analysis workflows.

In this work, we introduce a robotic platform designed to automate the collection of electrochemical reaction rate data collection. Our robotic system automatically performs queued electrochemical experiments that, unlike previous approaches, can accommodate different electrode, electrolyte, and gas-phase reactant composition for each individual experiment. This extent of operational versatility is not necessary for high-throughput catalyst screening but is essential for the kinetic analysis we sought to automate, which involves testing fresh electrocatalysts under a wide range of different operating conditions. We used this system to investigate the mechanism of CO_2_RR to carbon monoxide (CO) at several immobilized metal tetrapyrroles including cobalt phthalocyanine (CoPc), cobalt tetraphenyl porphyrin (CoTPP), and iron phthalocyanine (FePc). The mechanism of CO_2_RR at these catalysts remains a topic of debate,^16,30−42^ despite the fact that they are commonly studied and present relatively well-defined active site structures. We show that across these catalysts, nonidealities in reaction rate data are prevalent, specifically evidenced by bicarbonate and CO_2_ order dependencies that, in addition to being nonlinear and/or noninteger, also change with different applied potentials. We discuss how several reaction phenomena not commonly considered for these catalysts, such as electrolyte poisoning, mixed control, and coverage effects, can explain the trends. Our work demonstrates how automation can assist with cohesive kinetic analysis and sets forth general mechanistic considerations for interpreting complex rate data.

## 2 Results and Discussion

### 2.1 Overall Robot Design

**Figure 1:**
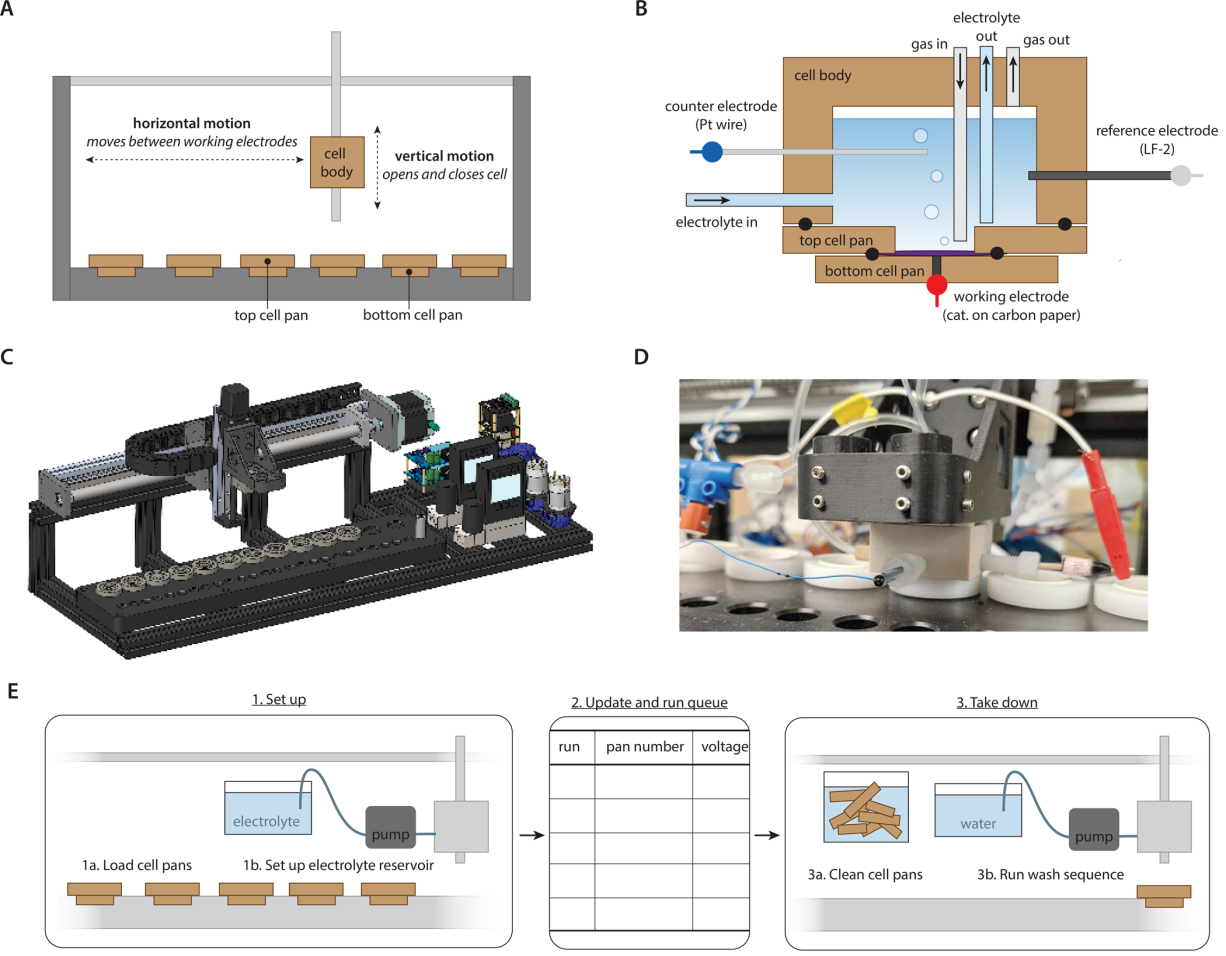
Robot design for the automated experiments. (A) Schematic of operation, where vertical motion opens and closes a cell body on top of a pair of cell pans and horizontal motion moves the cell body to different cell pans. Together these motions allow automatic execution of a different experiment at each cell pan. (B) Schematic of cell geometry, where the cell body houses ports for working and counter electrodes as well as ports for electrolyte/gas flow in/out of the cell. The top and bottom cell pan sandwich a working electrode, which is pressed into a conducting pin. Blue, red, and gray dots indicate connections to the potentiostat. Seals between cell body and pans are made by o-rings, depicted as black circles. (C) CAD depiction of the overall robot design. (D) Photograph of the robot during operation, while an electrochemical experiment is being executed at one of the cell pans. (E) Schematic illustrating the operational workflow of the automated system.

The overall robot design consists of a single cell body that houses the reference and counter electrodes, complemented by a line of 10 cell pan pairs that each house a carbon paper working electrode. The cell body moves horizontally to sequentially perform electrochemistry on each cell pan and vertically to open and close itself onto any given cell pan (Figure 1A).

For any single electrolysis within a given cell pan, the robot closes the cell body onto the cell pan and then pumps the electrolyte liquid through an “electrolyte in” port on the side of the cell body (Figure 1B). There are two possible configurations for the electrolyte source: each cell pan can have its own individual electrolyte that is stored locally in a nearby vial (Figure S2A), or the electrolyte source can be connected to one common reservoir (Figure S2B), so that a given set of electrolyses uses the same electrolyte formulation. In this work, the second option was employed, so that the electrolyte reservoir could be freshly bubbled with CO_2_. After electrolyte is flowed into the cell, the robot initiates gas flow through the “gas in” tubing (Figure 1B) and then applies voltage and initiates online product quantification. A gas chromatograph connected to the “gas out” port confers the online detection of CO and H_2_ (Figure 1B). Electrolyses were run for 40 min, and reported rates are averages of data collected between 20 and 40 min of electrolysis. We confirmed no systematic increases or decreases in the reaction rate within this time window (Figure S5). Finally, when the electrolysis is complete, electrolyte is pumped out of the cell and into a waste vial or container via the “electrolyte out” tubing and the cell is opened, completing the run.

Cell body cleaning is achieved by moving and closing the cell body onto a wash station consisting of an empty cell pan pair and then pumping milli-Q water in and out for several cycles (Figure S3). In this work, with the common reservoir electrolyte configuration, the cell body was rinsed after the completion of a full set of 10 electrolyses. We expected that this would not cause contamination issues because the same electrolyte composition was used within any given set of electrolyses and all products were gas-phase.

### 2.2 Discussion of Cell Design and Data Robustness

The cell assembly is analogous to a three-electrode, undivided compartment cell (Figure 1B). A carbon paper disk working electrode is clamped between a top and bottom cell pan, which forces it into electrical contact with a conductive rod and exposes a well-defined 10 mm diameter circle to the electrolyte. A leak-free Ag/AgCl reference electrode (Innovative Instruments) is inserted on the side of the cell body, and a 0.25 mm diameter platinum wire counter electrode is inserted on the opposite side of the cell body. We opted for this cell design because it uses the same electrode form factor as conventional electrochemical sandwich cell testing and the lack of membrane allowed for more facile liquid and gas management.

We note that as a one-compartment, rather than a two- or three- compartment cell, our setup comes with some considerations. First, without a membrane to separate the anode and cathode, there can be crosstalk between working and counter electrodes. For example, oxygen evolved at the counter electrode can cross over to the working electrode and lead to a parasitic oxygen reduction reaction (ORR) current. We did in fact observe incomplete Faradaic efficiency (FE) closure, where across all runs, an average of only ca. 60% was attributable to CO and H_2_ formation, and the remaining Faradaic efficiency was presumably lost to oxygen reduction (Figure S6). Additionally, we opted to use a Pt, rather than carbon-based, counter electrode to preclude any possibility of attributing adventitious anode oxidation products to CO_2_RR. Metal crossover from Pt anodes onto copper CO_2_RR cathodes has been observed, even in divided cells, to enhance hydrogen evolution.^43^ We did in fact observe relatively high FEs toward HER, typically around 30% to 60% (Figure S12). However, for dispersed molecular catalysts with relatively low surface coverage, we expected this effect to have a minimal impact on the kinetic interpretation of the partial CO current, which occurs on metal tetrapyrrole sites and should thus be relatively independent of any adventitious HER sites caused by Pt deposition. Finally, while many electrochemical CO_2_RR cell designs feed CO_2_ directly to or through the working electrode for improved mass transport, we bubbled CO_2_ into the electrolyte. We note that the currents in this study are below the theoretical mass transport limited current density (Supplemental Discussion 3.2) and that we did not observe an increase in partial CO current when increasing the flow rate beyond the 20 sccm that was used in this work (Figure S8). However, we acknowledge that flow configuration can have unexpected influence over measured kinetics, even in ostensibly non-transport-limited regimes.^44^

Given the above considerations, we first evaluated data robustness by using the automated setup to collect data that we could compare with literature precedent. We collected kinetic data for immobilized CoPc and compared it against previously reported results^16^ that were manually collected in a more classic three-compartment cell. We found that the newly measured rates toward CO production were up to 10 times lower than previously reported (Figure S10) and that Faradaic efficiencies toward CO were lower than expected, with values typically under 30% (Figure S12A). Nonetheless, we did find that previously reported kinetic trends in CO production were preserved (Figure S9) and that the newly collected data were well-correlated with those of the previous work (Figure S10). Importantly, hallmarks of nonideal reaction mechanisms, such as curvature and potential-dependent changes in the bicarbonate order dependence, were clearly evident in the data. Thus, we proceeded to analyze CoTPP and FePc to determine whether signatures of nonideal reaction mechanisms could be more broadly observed at other immobilized tetrapyrroles. Aggregated rates (Figure S11) and selectivities (Figure S12) toward CO and H_2_ for all three catalysts are provided in the Supporting Information.

### 2.3 Complex Kinetic Behaviors across CoPc, CoTPP, and FePc

We analyzed the partial currents toward CO production at different applied voltages, CO_2_ partial pressures, and bicarbonate concentrations. For each of these variables, signatures of mechanistic complexity, such as nonlinear and/or condition-dependent trends, were apparent in the corresponding canonical Tafel, CO_2_, and bicarbonate dependences.

**Figure 2:**
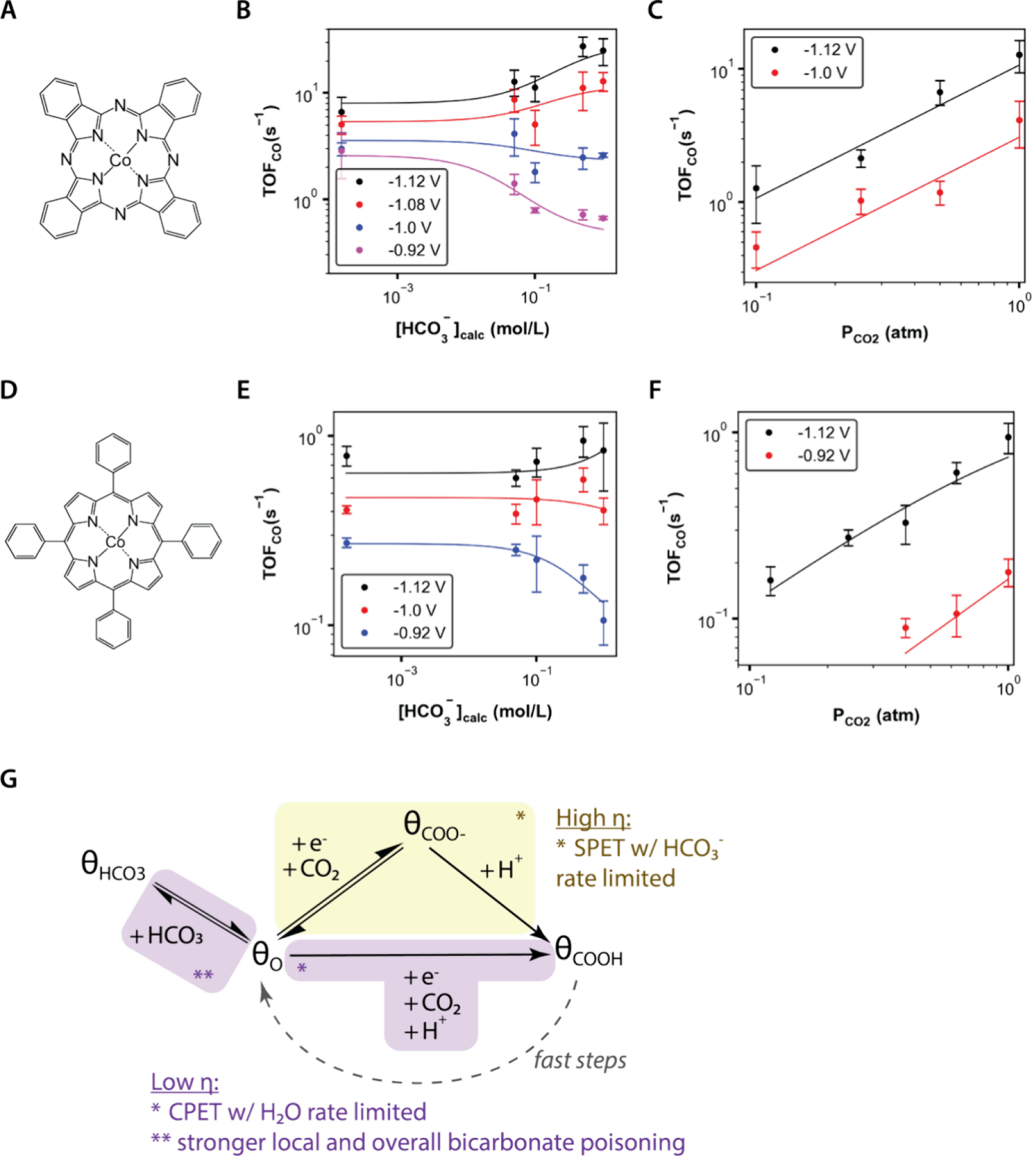
Kinetic analysis of Co-based tetrapyrroles. (A, D) Structures of catalysts studied: cobalt phthalocyanine (CoPc, A) and cobalt tetraphenyl porphyrin (CoTPP, D). (B, E) Apparent bicarbonate order dependencies at different voltages for CoPc (B) and CoTPP (E). (C, F) Apparent CO_2_ order dependencies at different voltages for CoPc (C) and CoTPP (F). Points represent experimental data, and lines represent model fits. Error bars represent standard deviation with *n* ≥ 3. For all experiments, voltages reported vs SHE, total ionic strength held constant using NaClO_4_, and CO_2_ at 1 atm. (G) Mechanistic framework that can explain the kinetic observations. Salient phenomena at less reductive voltages are displayed in purple, and salient phenomena at more reductive voltages are displayed in yellow.

**Figure 3:**
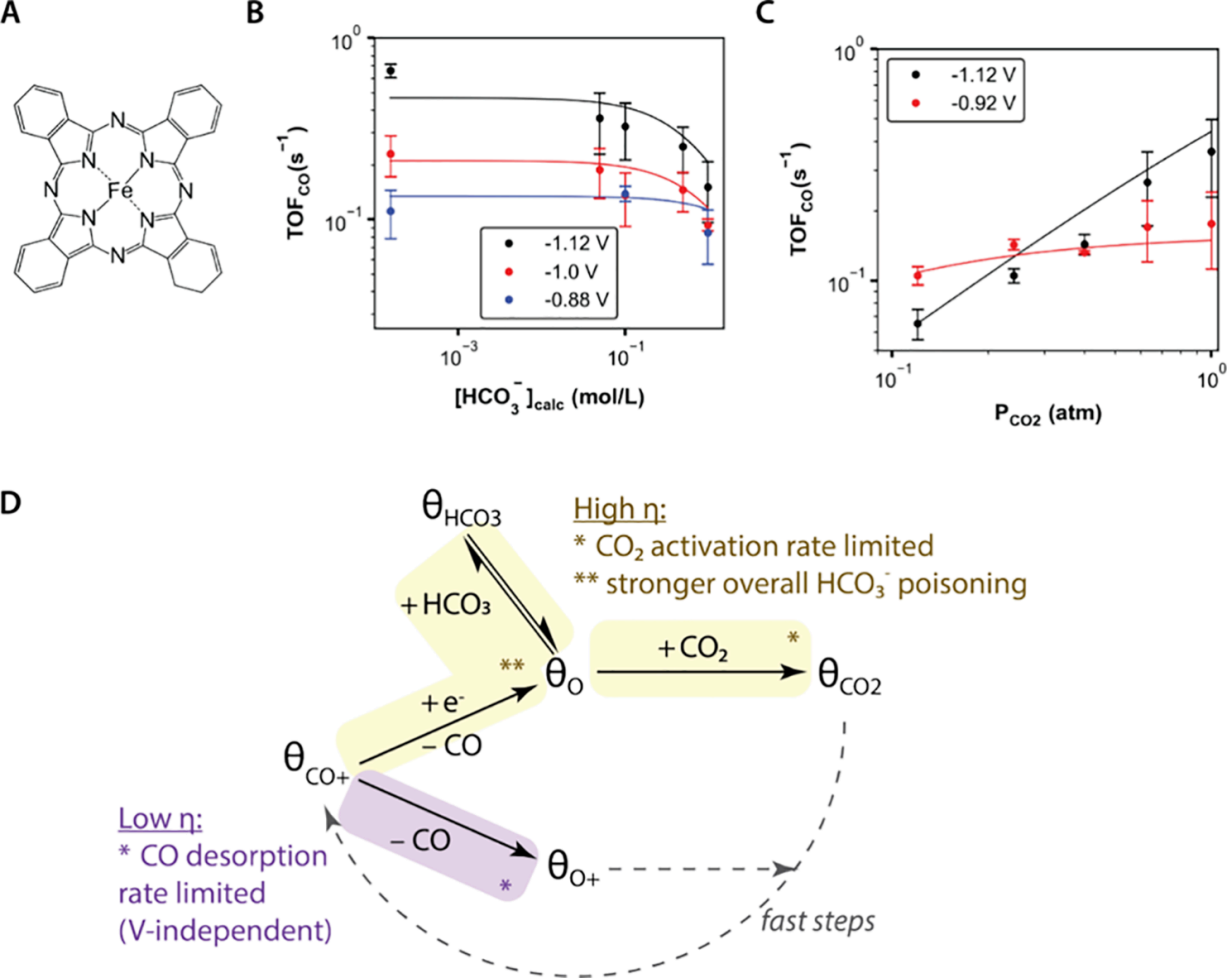
Kinetic analysis of Fe-based tetrapyrrole. (A) Structure of catalyst studied: iron phthalocyanine (FePc, A). (B) Apparent bicarbonate order dependencies at different voltages. (C) Apparent CO_2_ order dependencies at different voltages. Points represent experimental data, and lines represent model fits. Error bars represent standard deviation with *n* ≥ 3. For all experiments, voltages reported vs SHE, total ionic strength held constant using NaClO_4_, and CO_2_ at 1 atm. (D) Mechanistic framework that can explain the kinetic observations. Salient phenomena at less reductive voltages are colored purple, and salient phenomena at more reductive voltages are displayed in yellow.

The three tested catalysts present two general trends in the kinetic data, with the cobalt-based tetrapyrroles (CoPc and CoTPP) displaying qualitatively distinct features from the iron-based tetrapyrrole (FePc). For the bicarbonate dependence, all three catalysts display voltage-dependent bicarbonate inhibition. However, for CoPc and CoTPP, greater bicarbonate inhibition occurs at less reductive potentials (Figure 2B and 2E), whereas for FePc, the opposite is true (Figure 3B). For the CO_2_ dependence, FePc displays attenuation of the apparent CO_2_ order (*n*_CO2_ = 0.2 ± 0.1) at less reductive potentials, whereas CoPc and CoTPP do not display significant deviations from apparent CO_2_ orders of 1.

Such kinetic signatures have, to our knowledge, only been reported in one previous work at CoPc^16^ and are unexplainable with mechanisms typically invoked for CO_2_RR at immobilized tetrapyrroles.^30,32,34,37−39,42,45−49^ Below, we discuss the mechanistic features that likely underpin the observed trends. For the sake of clarity, the following discussions center around specific mechanistic proposals. However, we note that these mechanisms only qualitatively capture the observed trends but do not quantitatively, via statistical goodness-of-fit metrics, fit all of the experimental data. Thus, rather than proposing specific reaction mechanisms, the following discussion is mainly intended to (1) highlight the unambiguous existence of mechanistic complexity in the rate data at CoPc, CoTPP, and FePc and (2) describe the likely roles of phenomena such as mixed rate control, surface coverage effects, and catalyst poisoning in explaining the complex experimental behavior. Rate law derivations for the presented models are provided in SI Section 5.2, and examples of alternative models that were considered are provided in SI Section 5.1.

### 2.4 Mechanistic Features Proposed for CoPc and CoTPP

For CoPc and CoTPP, nonideality in the reaction rate data is most apparent for the bicarbonate dependence, where bicarbonate inhibits the reaction at less reductive potentials (low overpotential, or η) but displays a positive dependence at more reductive potentials (high η).

These trends can be explained by a mechanism that invokes two key features: (1) voltage-dependent bicarbonate poisoning and (2) voltage-dependent mixed control between bicarbonate-dependent and bicarbonate-independent reaction pathways (Figure 2G). Such a model has previously been reported to quantitatively describe the kinetic behavior at CoPc^16^ and can qualitatively capture observed trends for CoPc and CoTPP in this work, where the solid curves in Figure 2B, 2C, 2E, and 2F represent model fits.^16^

The first feature, bicarbonate poisoning, is modeled with a positive electrosorption valency, meaning that the negative charge on bicarbonate becomes more repelled from (or less attracted to) the surface as the electrode becomes more negatively (or less positively) charged during reductive polarization. Thus, at more reductive potentials (high η values), the local poisoning equilibrium shifts away from θ_HCO3_, which decreases the extent of bicarbonate inhibition.

The second feature, mixed control, is modeled as two concurrent reaction pathways involving a concerted proton–electron transfer (CPET) and sequential proton–electron transfer (SPET). At low η, the CPET with water as a proton donor is dominant, and at high η, the SPET with bicarbonate and water as proton donors becomes more dominant. Thus, at high η, bicarbonate becomes a more kinetically important proton donor, which paired with a decrease in bicarbonate inhibition (*vide supra*), leads to a positive apparent order in bicarbonate.

We note that the proposed mechanism can also account for apparent attenuation in CO_2_ order dependence at high η, which has previously been observed at CoPc.^16^ This phenomenon occurs because of coverage effects, where high η leads to accumulation of the surface species θ_COO–_. However, for the conditions tested in this work, the apparent orders in CO_2_ for CoPc and CoTPP remain within error of 1.

### 2.5 Mechanistic Features Proposed for FePc

For FePc, nonideality is apparent in both the bicarbonate and CO_2_ dependencies. For the bicarbonate dependence, inhibition increases at more reductive potentials (high η), and for the CO_2_ dependence, the apparent CO_2_ order becomes attenuated at less reductive potentials (low η).

These trends can be explained by a mechanism that invokes two key features: (1) voltage-dependent bicarbonate poisoning involving the interplay of two elementary steps and (2) voltage-dependent mixed control between CO desorption and CO_2_ activation (Figure 3D). One major difference between the model for FePc and that for CoPc and CoTPP is that the most abundant reactive intermediate (MARI), or resting state, of the catalyst is now a CO-adsorbed catalyst site (θ_CO+_) rather than an empty catalyst site (θ_O_). This agrees with the chemical intuition that Fe tends to bind CO more tightly than Co does. The model qualitatively captures observed trends for FePc, where the solid curves in Figure 3B and 3C represent model fits.

The first feature, bicarbonate poisoning, must still be modeled with a positive electrosorption valency, meaning that locally, the equilibrium between θ_HCO3_ and θ_O_ disfavors θ_HCO3_ at high η. While this local picture is in ostensible contrast to what is observed experimentally, the contradiction is resolved by accounting for the fact that MARI is a CO-adsorbed site (θ_CO+_). Thus, the coverage of θ_HCO3_ is governed by the interplay between two elementary steps: the local bicarbonate adsorption mentioned above and the voltage-dependent desorption of CO to convert θ_CO+_ to θ_O_. Since the latter step is favored by high η and has a stronger voltage dependence, the overall effect is to increase the coverage of θ_HCO3_ and observe stronger bicarbonate inhibition at high η.

The second feature, mixed control, is modeled as two concurrent pathways, where the voltage-independent desorption of CO is rate limiting at low η and mixed control between voltage-dependent CO desorption and CO_2_ adsorption are rate limiting at high η. Thus, at low η, because CO desorption is CO_2_-independent, the rate shows an attenuated dependence in CO_2_. Then, at high η, CO desorption becomes favored by reductive polarization, and CO_2_ activation becomes more rate limiting, causing the apparent dependence in CO_2_ to become first order.

This model also provides intuition for why the CO_2_ order dependence curves at low and high η intersect, which, alternatively stated, means that at low CO_2_ partial pressures, voltage inhibits the reaction. In the model, voltage directs reaction flux toward the accumulation of θ_O_, which is an unproductive pathway at low CO_2_ partial pressures because the subsequent CO_2_-dependent elementary step that converts θ_O_ to θ_CO2_ is sluggish. However, at higher CO_2_ partial pressures, the CO_2_-dependent pathway becomes faster, which causes the pathway to become productive and alleviates the inhibitory effect of voltage.

## 3 Conclusions

In this work, we report a robotic system that enables flexible and automated data collection for heterogeneous electrocatalysis. The resulting experimental workflow was more time- and labor-efficient, enabling faster and more facile rate data collection over a wide range of reaction conditions for several common CO_2_RR electrocatalysts. Analysis of these data using cohesive kinetic analysis highlighted hallmarks of mechanistic complexity not interpretable via common Tafel or order dependence analyses.

Specifically, across immobilized CoPc, CoTPP, and FePc catalysts, we report CO_2_ and bicarbonate order dependences that are nonlinear and/or change depending on the applied voltage. We describe how these behaviors can be explained by models that invoke bicarbonate poisoning and a potential-dependent mixed control. We note that beyond an initial discussion of these phenomena at CoPc,^16^ such kinetic behaviors are not discussed for CO_2_RR at immobilized metal tetrapyrroles. In the existing mechanistic debates surrounding this class of electrocatalysts, only steps such as CO_2_ adsorption accompanied^30,32,33^ or preceded^34,35^ by electron transfer, CO_2_ adsorption accompanied by concerted proton electron transfer,^36,37,41^ CO* formation,^50^ CO* desorption,^39,40,46,51^ and discrete metal redox events^38−40,52^ are among those typically asserted and/or debated. Although the likelihood of voltage-dependent mechanistic changes is acknowledged,^34,41,42^ it is seldom experimentally interrogated. Our work highlights that kinetic phenomena such as electrolyte poisoning, coverage effects, and mixed control are important considerations for the CO_2_RR at metal tetrapyrroles. Additionally, our work suggests that ostensibly opposing kinetic observations in the literature might be reconciled by differences in operating conditions, where kinetic measurements reported in a limited range of operating conditions only “see” one part of a much more complicated whole.

Finally, future work extending this initial robotic concept could explore incorporation of a membrane to separate the anode and cathode, as well as alternative electrolyte and gas flow configurations. Additionally, software development to automatically enumerate mechanisms and suggest the most informative experimental conditions could further standardize and accelerate the workflow. We anticipate that, with further engineering efforts, extensions of this automated system may not only improve the throughput and reduce the labor requirements of data collection but also improve reliability and consistency of the data itself. Our work provides an initial demonstration of how the convergence of automation with cohesive kinetic analysis provides an informative experimental strategy for dissecting electrocatalytic reaction complexity.

## 4 Methods

### 4.1 Electrode and Electrolyte Preparation

CoPc (Sigma-Aldrich) catalysts were dissolved in *N*,*N*-dimethylformamide (DMF) and drop-casted onto calcined Toray060 carbon paper (Fuel Cell Store) electrodes at loadings of 5.98 × 10^–11^ mol/cm^2^. CoTPP (Frontier Scientific) and FePc (Sigma-Aldrich) catalysts were dissolved in carbon black-containing DMF solutions and drop-casted at loadings of 5.11 × 10^–10^ and 8.04 × 10^–10^ mol/cm^2^, respectively. Carbon black was used for the less active catalysts to help increase catalyst loading while also avoiding aggregation.^53^ Thus, all catalysts were tested at low loadings where aggregation was not expected to be an issue.^54^

Electrolytes were made by preparing stock solutions of 0.5 mol/L Na_2_CO_3_ (Sigma-Aldrich) and 1 mol/L NaClO_4_ (Sigma-Aldrich). Upon preparation, these stock solutions were bubbled overnight with CO_2_ and stored for further use. Electrolyte solutions with overall 1 mol/L ionic strength were then prepared by mixing the stock solutions in appropriate volumetric ratios to achieve desired bicarbonate concentrations.

### 4.2 Robot Operation

Typically, electrolyses were queued in batches of 10, with all 10 electrolyses using the same electrolyte composition. Working electrodes were loaded into cell pans, and the electrolyte reservoir was filled with electrolyte and bubbled with CO_2_. The Pt wire counter electrode resided in the cell body and was not often removed. The leak-free Ag/AgCl reference electrode (Innovative Instruments) was calibrated against a saturated calomel electrode every day and was swapped out every 1–3 days. After queueing and running the 10 electrolyses, the cell body washing sequence would be executed and the cell pans would be washed manually.

### 4.3 Data Handling

All data files, including electrolyses and reference calibrations, were automatically uploaded to an SQL database. A Python script was used to access files with relevant run conditions. It then output average current, partial CO current, partial H_2_ current, and operating conditions to an Excel document.

Throughout this work, no unexpected or unusually high safety hazards were encountered.

## Supporting Information Available

Reagent information, detailed experimental protocols, supplemental data, and additional discussion (PDF)
Transparent Peer Review report available (PDF)
All experimental data reported in this work (XLSX)

## ABBREVIATIONS

CO_2_RR: carbon dioxide reduction reaction
CoPc: cobalt phthalocyanine
CoTPP: cobalt tetraphenyl porphyrin
FePc: iron phthalocyanine
FE: Faradaic efficiency
HER: hydrogen evolution reaction
ORR: oxygen reduction reaction
TOF: turnover frequency
