# Supplementary material for: Nonidealities in CO2 Electroreduction Mechanisms Revealed by Automation-Assisted Kinetic Analysis
Source: ACS Cent Sci. 2024 Jun 28;10(7):1348–56. doi: 10.1021/acscentsci.3c01295 (PMC11273456; doi:10.1021/acscentsci.3c01295)
Supplement: Supplementary file 1 — oc3c01295_si_001.pdf [file oc3c01295_si_001.pdf]

Non-idealities in CO_2 electroreduction mechanisms revealed by automation-assisted kinetic analysis

Joy S. Zeng, [1,†] Vineet Padia, [2,†] Grace Y. Chen,[3] Joseph H. Maalouf,[1] Aditya M. Limaye,[1] Alexander H. Liu,[1] Michael A.Yusov,[3] Ian W. Hunter,[2] Karthish Manthiram[3,]*

[1]Department of Chemical Engineering, Massachusetts Institute of Technology, 77 Massachusetts Avenue, Cambridge, Massachusetts 02139, United States

[2]Department of Mechanical Engineering, Massachusetts Institute of Technology, 77 Massachusetts Avenue, Cambridge, Massachusetts 02139, United States

[3]Division of Chemistry and Chemical Engineering, California Institute of Technology, Pasadena, California 91125, United States

[†]Equal contribution, *Correspondence: karthish@caltech.edu

Contents

1. Materials & methods for the automated setup 2
   1.1 Robot parts 2
   1.2 Software control details 3
   1.3 Additional details about robot operation 4
   1.4 Abbreviated standard operating procedure (common electrolyte reservoir configuration) 5
2. Materials & methods for electrochemical testing 6
   2.1 Chemicals 6
   2.2 Electrode & electrolyte preparation 7
   2.3 Electrochemical testing 7
3. Additional discussion about cell design & data robustness 10
   3.1 Faradaic efficiency closure 10
   3.2 Mass transport and effects related to flow configuration 10
   3.3 Geometric effects 12
   3.4 Benchmarking CoPc against previous work 12
4. Aggregated rate data for CoPc, CoTPP, and FePc 13
   4.1 Rate 13
   4.2 Selectivity 14
5. Additional discussion about mechanisms 15
   5.1 Inventory of all models tested 15
      5.1.1 Models for CoPc and CoTPP 15
      5.1.2 Explaining kinetic trends at FePc 18
   5.2 Derivation of rate laws for proposed mechanisms 21
      5.2.1 Rate law for CoPc and CoTPP (Model A-1) 21
      5.2.2 Rate law derivation for FePc (Model B-3) 23

1. Materials & methods for the automated setup

1.1 Robot parts

Electronic controls (from Tinkerforge)

- Master Brick (x1)
- Silent Stepper Brick (x 1)
- Silent Stepper Bricklet (x 2)
- RS232 Bricklet 2.0 (x 2)
- Industrial Quad Relay Bricklet 2.0 (x 4)
- Bricklet Cables, assorted lengths

Power supply

- Buck Boost Converter (Dmyond ZK-DP60 or DROK zk-sjva-4x)

Actuators (stepper motors)

- Fuyu FSL30 Mini Linear Stage Actuator (150 mm stroke)
- Fuyu FSL40 Linear Guide (400 mm stroke)

Pumps

- Kamoer low flow peristaltic pump 12V DC 3mm ID x 5mm OD (x2)
- Kamoer small peristaltic pump stepper motor 24V Adjustable Speed KPAS-100, 110ml/min, 3.2mm ID ×6.4mm OD, 3 rotors (x 1)

Physical/structural components

- Misumi aluminum extrusions (25x25 Aluminum Extrusion - 5 Series, Base 25)
- Soft Viton o-rings (McMaster-Carr 1284N12 & 1284N116)
- Stainless steel dowel pins (McMaster-Carr 91585A361)
- Cable chain (Befenybay)
- Needle fittings (Idex P-675)
- Hxchen M3 x 12mm + 6mm Male to Female Thread Brass Hexagon Hex Standoff Spacer Pillars

The cell body (Figure S1A and Figure S1C) and cell pans (Figure S1D), as well as the mount for the cell body (Figure S1B) and next for the cell pans (Figure S1D), were custom-made parts. CAD files are available upon request.

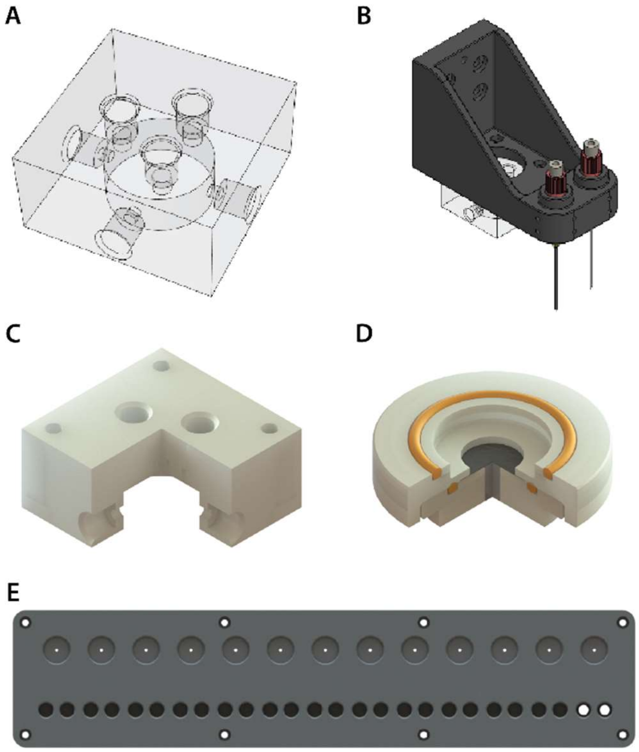

Figure S1. Illustrations of custom-made parts for the robotic assembly. **A**, cell body. **B**, mount for cell body and electrolyte needles. **C**, opaque cutout of cell body. **D**, opaque cutout of top and bottom cell pan. **E**, nest for cell pans and electrolyte vials.

1.2 Software control details

Control over the potentiostat was achieved using the PyExpLabSys package that utilizes the EClab Development Package. All code was implemented in 32-bit Python 3.8. Databasing was performed with PostgreSQL.

To queue up runs, the user enters pertinent run information such as cell pan number, catalyst identity, bicarbonate concentration, gas flow rate, CO_2 partial pressure, operating voltage, and electrolysis time into an Excel file. Then, the user runs a python driver script that reads in run information and physically controls the robot to execute the desired electrolysis conditions. Upon conclusion of the run, the script generates a timestamp-named folder and copies all pertinent files including current output and GC chromatogram files, into the folder. Then, the script populates a database with the run information and file path to the corresponding data folder. OCV measurements can also be queued up this way for reference electrode calibrations.

To process data from the database, query scripts can be used. Our analysis script automatically averaged current and GC data over a user-defined period of time (20 to 40 minutes) to output average partial currents, TOFs, and FEs for the different operation conditions. For electrode referencing, the script automatically selects the OCV measurement closest in time to any given run.

1.3 Additional details about robot operation

Electrolyte configurations

The robot is able to accommodate two electrolyte configurations, illustrated in Figure S2. The individual electrolytes configuration allows each run to have a different electrolyte formulation (Figure S2A). In this configuration, electrolyte is stored in parafilm-covered 1.5 mL GC-MS vials. If fresh gas bubbling is required, this must be achieved with an extended pre-electrolysis bubble after the electrolyte is pumped into the cell. The common reservoir configuration (Figure S2B) allows one to continuously bubble the electrolyte reservoir with gas, but requires one to manually change out the electrolyte reservoir when a different electrolyte is desired. In this work, we opted to use the common electrolyte reservoir method.

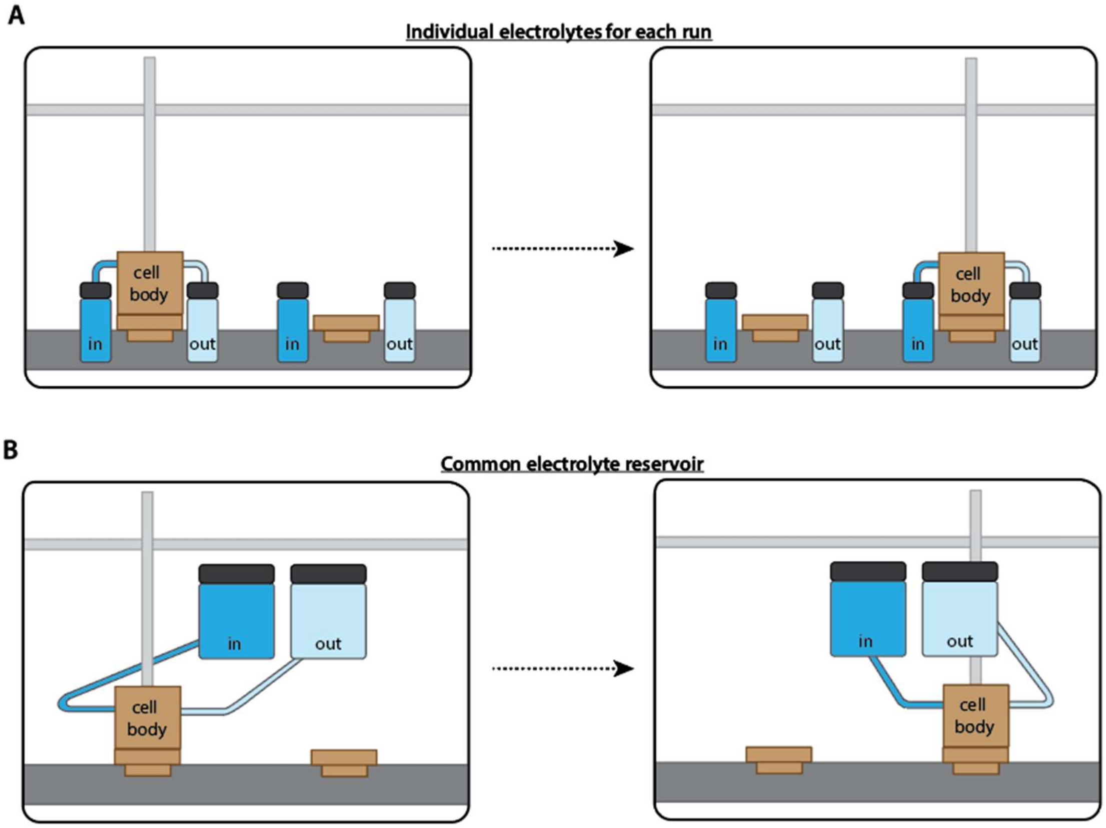

Figure S2. Electrolyte configuration schematics. **A**, setup for allowing individual electrolytes for each run. **B**, setup for allowing use of a common electrolyte reservoir.

Cleaning protocols

To rinse out the cell, the setup includes a "cleaning station", which is just a top and bottom cell pan with no carbon paper sandwiched inside. Next to the empty cell pans are two wash vials. The inlet wash vial is connected to a milliQ water source, where water from the source is freshly pumped into the vial and then subsequently pumped (with a separate pump) into the cell. The outlet vial is connected to a waste container (Figure S3). The cell is closed onto the cleaning station, filled with milliQ and then drained. The fill-drain cycle is repeated five times and then the cell is opened. For the common electrolyte reservoir configuration used in this work, this cleaning protocol was executed after every 10 electrolyses

or upon changing the electrolyte composition. Executing the wash sequence required one to manually move inlet tubing from the electrolyte reservoir to the wash vial. Cell pans were manually cleaned, and approximately every 24 hours, the reference electrode was swapped out and allowed to let sit in a storage solution. Every now and then, the bottom surface of the cell body would also be manually wiped down.

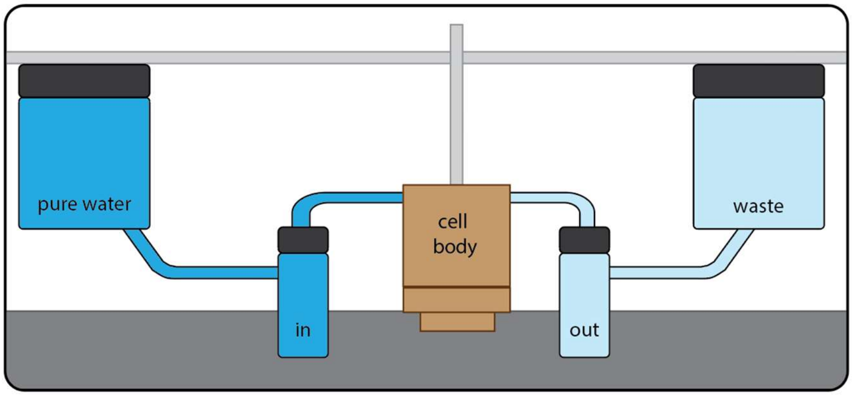

Figure S3. Wash station configuration.

1.4 Abbreviated standard operating procedure (common electrolyte reservoir configuration)

1. Calibrate reference electrode
    a. Recommended to calibrate reference every morning
    b. Set up leak-free reference with a master reference electrode (SCE or Sat Ag/AgCl) in a beaker/vial of sat'd KCl and clip potentiostat onto this setup (manually)
    c. Go into excel queue and add an entry for an OCV run
    d. Run the python script to execute the queue and database the OCV run
2. Physically set up the robot
    a. Rinse reference electrode and plug into the robot cell body, connect to reference clip
    b. Plug WE, CE, and RE leads of the potentiostat into the back of the robot
    c. Prepare desired electrolyte solution, put in a beaker or vial and bubble continuously with CO_2
    d. Put electrolyte inlet tubing into above electrolyte solution, run the "electrolyte in" pump until all of the "electrolyte in" tubing leading to cell is filled with electrolyte
    e. Assemble electrodes and cell pans
        i. Put in bottom cell pan with small o-ring and central stainless steel pin
        ii. Put electrode on top of botton cell pan
        iii. Put top cell pan + o-ring on top of electrode
        iv. Repeat this assembly for all 10 cell pan pairs
3. Set up software components
    a. Open PeakSimple (SRI GC control software), load appropriate control files, confirm results file naming conventions, and set post run to repeat the correct number of times required for a single electrolysis
    b. Update the excel queue with desired CA runs. Within the queue, the user specifies pan number, voltage, CO_2 partial pressure, CA duration, and total gas flow rate for the run, which the robot will read and set accordingly. Other parameters that the user sets, such as bicarbonate concentration and catalyst identity/loading should also be specified in the queue so that this information can be databased with the run.

   c. Run the python script to execute the queue and database the electrolyses. The queue can be continuously updated even while the robot is running.
4. Accessing data
   a. For each run, a timestamped folder will automatically be created within the runs directory. This folder contains a csv file with the electrochemistry output, a copy of the queue at the time of the run, a configuration file specifying details of the run, and GC files with CO and H_2 quantification data.
   b. All of this information is also uploaded to a PostgreSQL database.
5. Running wash sequence to clean the cell body
   a. Move the electrolyte pump inlet tubing to the wash station.
   b. Also recommended to use a moist kimwipe to gently pat down the bottom of the cell body, as sometimes salt deposits will accumulate.
   c. Make sure a bottom and top cell pan, as well as metal pin (electrode not necessary) are loaded in location 0
   d. Run the wash_cell method as many times as necessary (usually ~ 5 times)
6. Cleaning up
   a. Clean out the cell pans - remove all cell pans from the setup, discard carbon papers, wipe with kimwipes if there is carbon residue, and rinse+sonicate cell pans several times with milliQ. Wipe down to dry before using again.
   b. Put reference electrode back in storage if done for the day. If using multiple days continuously, recommended to switch between references every 1-2 days.

2. Materials & methods for electrochemical testing

2.1 Chemicals

All chemicals and materials were used as received unless otherwise specified.

Catalysts

- Cobalt(II) phthalocyanine (97%, Sigma Aldrich 307696)
- Iron(II) phthalocyanine (90%, Sigma Aldrich 379549)
- Cobalt(II) tetraphenyl porphyrin (97%, Frontier Scientific T40823)
- Toray 060 carbon paper (Fuel Cell store, 5% wetness proofing)
- Nafion 117 solution (5% in alcohols and water, Sigma Aldrich 70160)
- Carbon black (Vulcan XC 72, Fuel Cell Store)
- N,N-dimethylformamide (99.8%, Sigma Aldrich)

Electrolytes & electrochemical testing

- Sodium carbonate (>99.5%, Sigma Aldrich 223530)
- Sodium perchlorate (≥ 98.0%, Sigma Aldrich 410241)
- MilliQ water
- Carbon dioxide (99.999%, Airgas)

Electrode parts

- Leak-free Ag/AgCl reference electrode (Innovative Instruments LF-2)
- Pt wire, 0.25 mm diameter (99.9% (metals basis), Thermo Scientific Chemicals 045093-BU)

2.2 Electrode & electrolyte preparation

Electrode preparation was based closely on previous reports.[1,2] Briefly, circular electrodes with a diameter of 13 millimeters were punched from Toray 060 carbon paper and calcined in a muffle furnace in static air at 600 ^{o}C for 1 hour to generate hydrophilic carbon paper.

The electrodes for cobalt phthalocyanine (CoPc) were prepared by dispersing 10 mg of CoPc in 10 mL of N,N-dimethylformamide with 15 minutes of sonication. This solution was then serially diluted in DMF to obtain a final diluted ink with a concentration of 3.97 x 10^{-6} mol/L (faintly blue colored). 20 µL of this ink was then dropcasted onto a hydrophilic carbon paper electrode to yield a final CoPc loading of 5.98 x 10^{-11} mol/cm^2.

For the other two immobilized tetrapyrroles, cobalt tetraphenyl porphyrin (CoTPP) and iron phthalocyanine (FePc), inks containing carbon black (CB) and nafion were used to increase dispersion in increase catalyst loading. This increase in loading was required to help with product detection, since these catalysts displayed lower overall activity towards CO_2RR to CO. The base carbon black ink was prepared by adding 50 µL of 5 wt% Nafion solution to 20 mL of DMF. This nafion-containing solution was then mixed with carbon black in a ratio of 1 mg carbon black per 1mL of solution.

Then, the electrodes for CoTPP were prepared by dispersing 10 mg of CoTPP in 10 mL of the carbon black and nafion-containing solution. This solution was then serially diluted using the same CB+nafion DMF solution to obtain a final diluted ink with a concentration of 4.5 x 10^{-5} mol/L. 20 µL of this ink was then dropcasted onto a hydrophilic carbon paper electrode to yield a final CoTPP loading of 5.11 x 10^{-10} mol/cm^2.

The electrodes for FePc were prepared by dispersing 10 mg of FePc in 10 mL of the carbon black and nafion-containing solution. This solution was then serially diluted using the same CB+nafion DMF solution to obtain a final diluted ink with a concentration of 5.3 x 10^{-5} mol/L. 20 µL of this ink was then dropcasted onto a hydrophilic carbon paper electrode to yield a final CoTPP loading of 8.04 x 10^{-10} mol/cm^2.

2.3 Electrochemical testing

Electrolyte preparation

Stock 1 mol/L solutions of $NaHCO_3$ and $NaClO_4$ were prepared from Na_2HCO_3 and $NaClO_4$ powders and MilliQ ultra pure water. After mixing all components, both solutions were bubbled overnight with CO_2 before use. To prepare solutions of the desired $NaHCO_3$ concentration, these two stock solutions were mixed in the appropriate ratio, thus yielding electrolyte compositions of X mol/L $NaHCO_3$ and (1-X) mol/L $NaClO_4$. When in use, these solutions were continuously bubbled with CO_2 while stored in the electrolyte reservoir.

Electrolysis specifications

High-purity CO_2 gas was controlled by an Alicat mass flow controller and introduced into the cell at atmospheric pressure; CO_2 gas was bubbled at a flow rate of 20 sccm, using 1/16” OD tubing, near the surface of the working electrode. Upon exiting the cell, the gas flowed directly to an on-line gas chromatograph (SRI Instruments, Inc., MG #5, Model 8610C). CO_2 partial pressure was changed using N_2 gas as the balance. The working compartment was purged with CO_2 for 5 min before electrochemical polarization.

Electrolyses were performed with a Biologic SP-300 potentiostat, using chronoamperometry (CA) methods. 100% resistance compensation was applied manually in post-processing for each run, using a resistance of 15 ohms, determined via potentiostatic electrochemical impedance spectroscopy (PEIS) collected for a representative cell (Figure S4).

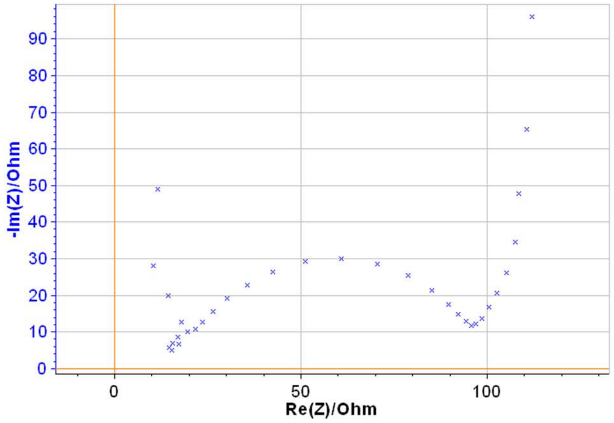

Figure S4. PEIS spectrum of a cell containing 1M $NaHCO_3$, showing a solution resistance of approximately 15 ohms.

Reference electrode calibration

The leak-free reference was calibrated every morning against a saturated calomel electrode, (CHI150, CH Instruments) in a saturated potassium chloride solution (The potential at the SCE was assumed to be, according the manufacturer specification, +0.241 V vs SHE at 25 ºC) via open circuit voltage measurements held for 120 seconds. The average OCV value over the last 90 seconds was used as the value, as long as the net drift over that time was less than 5 mV. The reference calibration for any run was taken to be that which was closest in time to the run.

Product quantification and rate calculation

The gas chromatograph was equipped with a thermal conductivity detector (TCD) and flame ionization detector (FID), a methanizer, and a Hayesep-D column. The FID was used to quantify CO and the TCD to quantify H_2. Every 10 minutes, sample flowing through the sample loop was flowed to the Hayesep-D column, where it flowed to the FID and TCD. CO_2 eluted after CO, and flow to the FID was diverted during the times at which CO_2 was expected to elute. Column temperature was maintained at 80 ºC. Electrolyses were performed for 40 minutes, and steady state partial currents were calculated based on the average of GC quantification at the 20, 30, and 40 minute marks.

We waited 20 minutes before beginning to use rate data so that the electrolysis would have ample time to reach a steady-state condition. After 40 minutes, we expected the electrolysis to be both long enough to average over several (three) GC injections, and short enough to reduce experimental time and mitigate potential issues with catalyst deactivation. The robustness of this approach is supported by the observation that across all runs, between 20 and 40 minutes, the reaction rates appear to be at steady state (Figure S5). Specifically, the "percent change" within this window, as calculated below, shows a normal distribution centered at 0, indicating no systematic increase or decrease of rates.

$$Percent\ change(j_{total}) = \frac{|avg\ from\ 35\ to\ 40\ mins| - |avg\ from\ 20\ to\ 25\ mins|}{|avg\ from\ 20\ to\ 25\ mins|}$$

$$Percent\ change(j_{CO}, j_{H2}) = \frac{(avg\ from\ 30\ to\ 40\ mins) - (avg\ from\ 20\ to\ 30\ mins)}{(avg\ from\ 20\ to\ 30\ mins)}$$

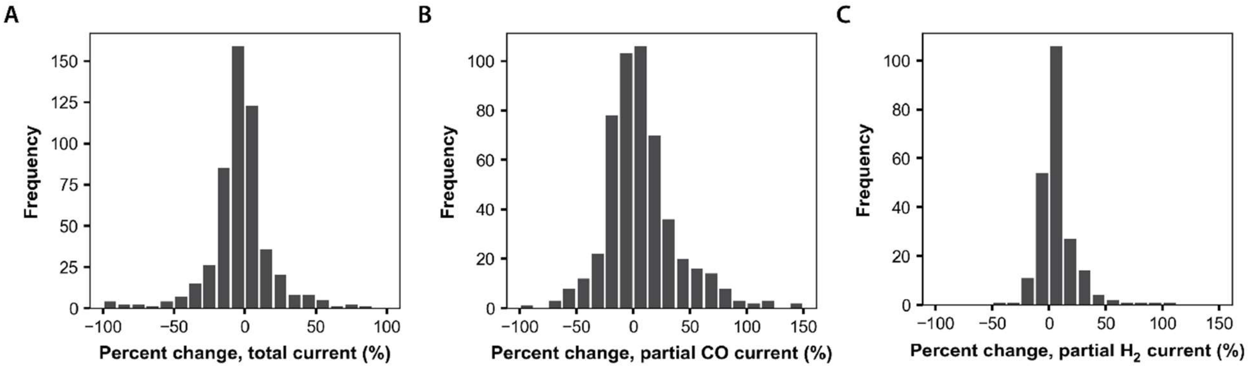

Figure S5. Histograms of percentage change in total current (**A**), partial CO current (**B**), and partial H_2 current (**C**), within the data collection period for all electrolyses reported in this work. In all three cases, the x-axis has been constrained for clarity; the number of points that lie outside of the shown plots are 3, 5, and 1 for panels **A**, **B**, and **C** respectively. Fewer points are shown in panel **C**, because runs with H_2 below the GC detection limit are not shown.

Finally, from this data, turnover frequency, defined as the reaction rate at each site, was calculated by assuming all deposited catalyst molecules were active. Thus, the formula for turnover frequency was:

$$TOF_{CO} = \frac{[CO] \cdot v_{out}}{\rho_{cat} \cdot A_{elec}}$$

Where $[CO]$ is the concentration of CO detected in the product stream in mol/cm^3, v_{out} is the flow rate of gas exiting the cell in cm^3/s, ρ_{cat} is the loading of catalyst deposited on the electrode in mol/cm^2 and A_{elec} is the active (exposed) area of the electrode, assumed to reflect the size of the hole in the upper cell pan.

3. Additional discussion about cell design & data robustness

3.1 Faradaic efficiency closure

Faradaic efficiency closure for the two expected products, hydrogen and carbon monoxide gas, were usually between 50-80%, which is low compared to the expected 100% (Figure S6). This is likely due to background ORR, possibly exacerbated by OER at the counter electrode of the 1 compartment cell.

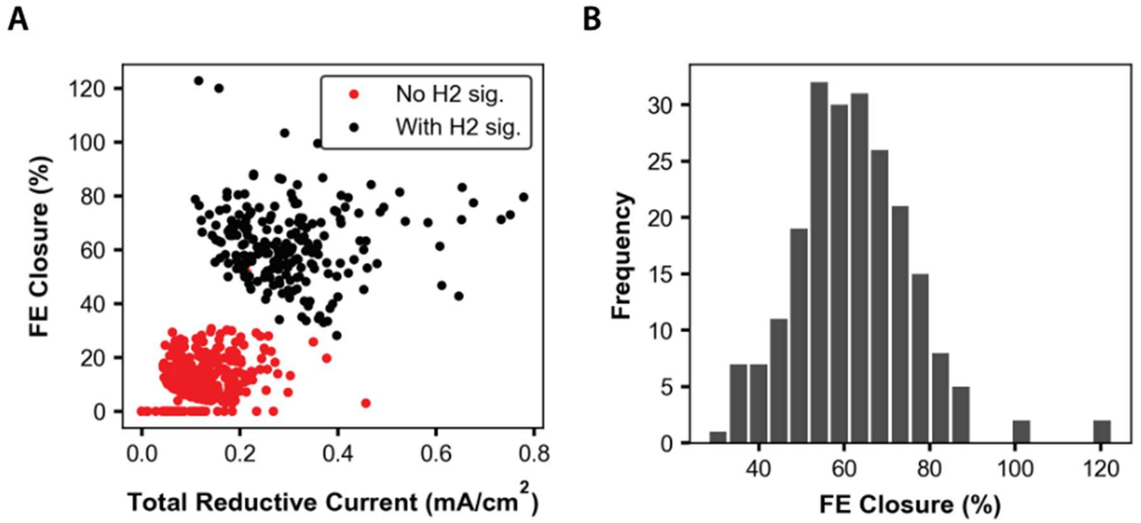

Figure S*6*. Faradaic efficiency closure for all runs reported. **A**, FE closure for all runs plotted as a function of total current density. Points that had detectable H_2 signal are shown in black, and points without detectable H_2 are indicated in red. **B**, histogram of the FE closures observed for runs in which both CO and H_2 were detectable.

3.2 Mass transport and effects related to flow configuration

To assess the importance of CO_2 mass transport limitations, we calculated the theoretical transport limited current density to a planar surface, given by:

$$i_{lim} = nF\frac{D_A C_A}{\delta}$$

Where n is the number of electrons transferred in the reaction, F is Faraday's constant, D_A and C_A are the diffusivity and bulk concentrations of CO_2, assumed to be the most limiting reactant in the system (we note that this is an assumption due to pH gradients that are likely to be present at the electrode surface), and δ is the hydrodynamic boundary layer thickness. CO_2 has a solubility of ~33 mM and a diffusivity of 1.91 x 10^{-5} in water.[3] We will assume a boundary layer thickness of about 200 μm, which has been shown in similar cells.[4] Taken together, this gives:

$$i_{lim} = 2 \cdot 96485\,\frac{(1.91E{-}5\frac{cm^2}{s})(33E{-}3\frac{mol}{L})(\frac{1L}{1000\,cm^3})}{2E{-}2\,cm} = 6\ \mathrm{mA/cm^2}$$

The kinetic data collected in this work are all below 0.5 mA/cm^2 (Figure S7) which is more than an order of magnitude lower than this value.

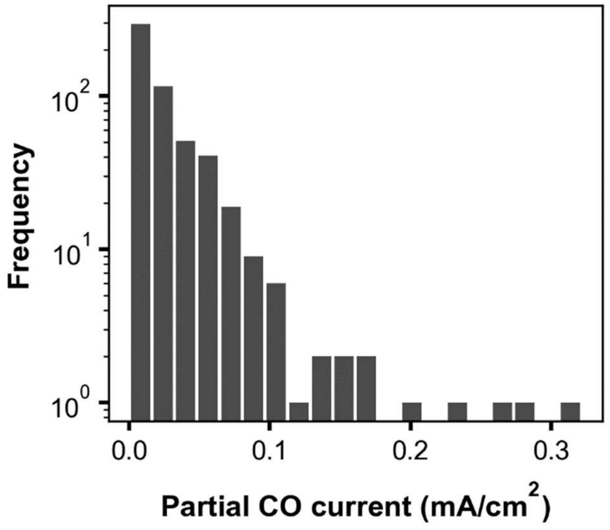

Figure S7. Histogram of partial reductive current densities toward CO production of all runs reported in this work. Notably, all values are well below the value of 6 mA/cm^2 that is the theoretical CO_2 transport-limited current density.

Additionally, to experimentally demonstrate transport-free kinetic control, we collected reaction rate data as a function of CO_2 flow rate. Since CO_2 bubbling in the cell is the source of electrolyte convection, we expected that increasing the CO_2 flow rate would also increase convection, which decreases boundary layer thickness and improves mass transport. Beyond the flow rate of 20 sccm that was used in this work, we observe no increase in rates with increased convection (Figure S8), experimentally suggesting the lack of mass transport limitations

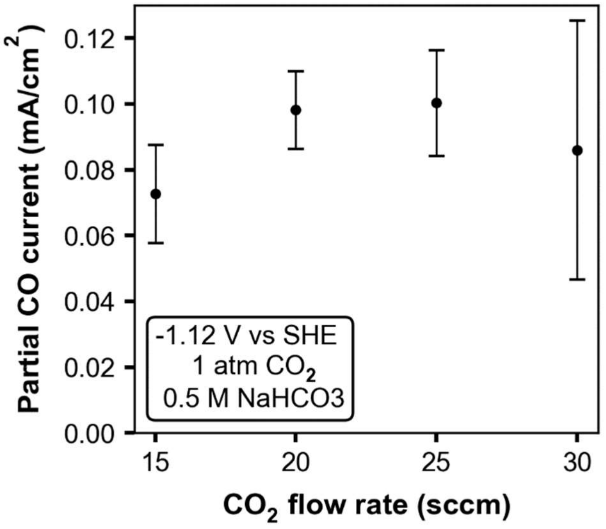

Figure S8. Partial CO current as a function of CO2 flow rate. Notably, beyond 20 sccm, the rate does not significantly change with flow rate. Error bars represent standard deviation with n = 2.

3.3 Geometric effects

With asymmetry between working and counter electrode, there can, in theory, be inhomogeneity in current flux at different locations on the working electrode. These inhomogeneities are more problematic for highly resistive solutions, and for the 1 M ionic strength solutions used here, this is not expected to be an issue.

3.4 Benchmarking CoPc against previous work

To get a better sense of data robustness, we first used the robot to reproduce kinetic data on CoPc that has already been reported (Figure S9).[2] We found that although the absolute values are lower, the non-ideal trends, particularly in the Tafel and bicarbonate dependences, are largely recapitulated. This suggested to us that the automated data collection would provide sufficiently high quality data to assess the presence of complex reaction mechanisms at other catalysts such as CoTPP and FePc. A correlation plot between previously reported manual data collection[2] and this work, for points collected at the same operating conditions, is shown in Figure S10.

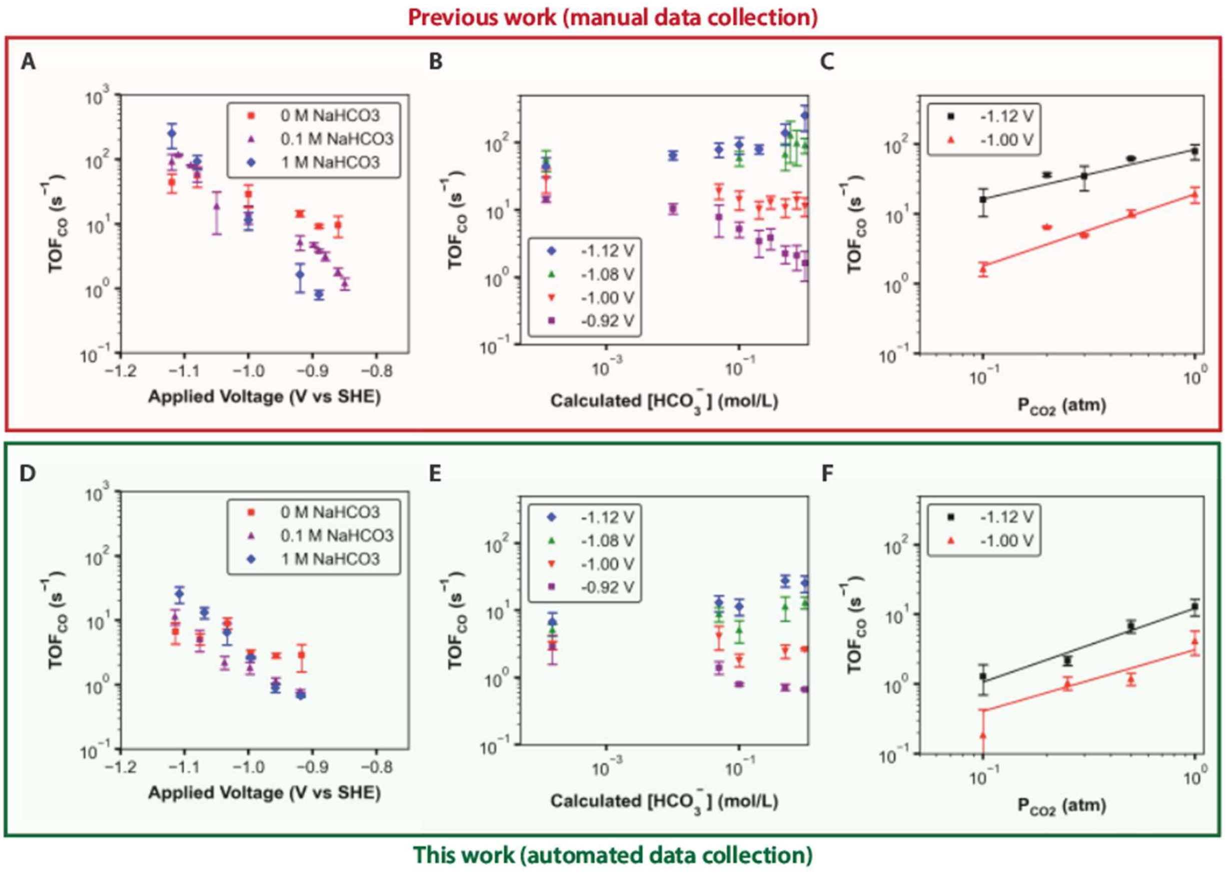

Figure S9. Benchmarking automated data collection against manual data collection at a CoPc catalyst. **A-C** were collected manually and reported in a previous work.[2] **D-F** were collected with the automated setup in this work. **A**, **D**: Tafel dependence at several bicarbonate voltages. **B, E**: bicarbonate dependence at several voltages. **C, F**: CO_2 dependence at several voltages.

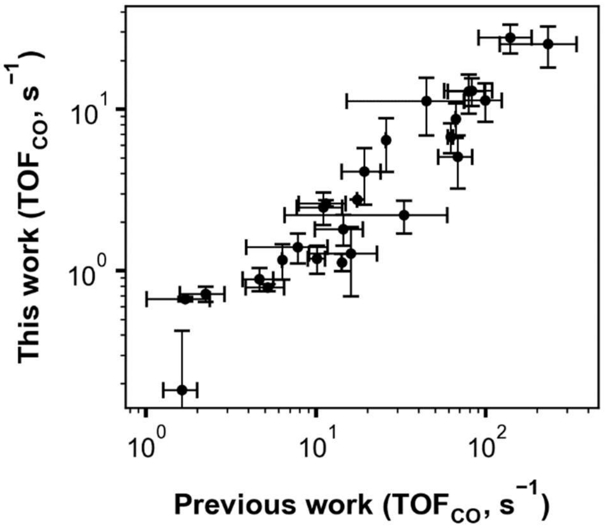

Figure S10. Correlation between CO rate data reported in a previous work[2] and that reported in this work.

4. Aggregated rate data for CoPc, CoTPP, and FePc

4.1 Rate

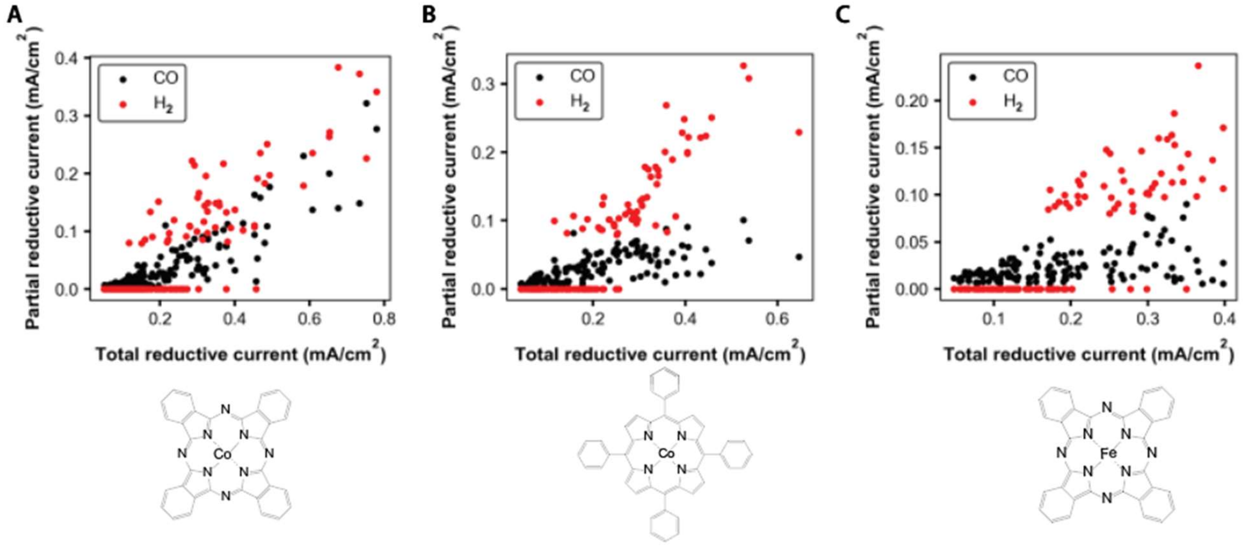

Figure S11. Partial reductive current towards both CO and H_2, aggregated across all runs based on total reductive current, for the three different catalysts. **A**: CoPc; **B**: CoTPP; **C**: FePc.

4.2 Selectivity

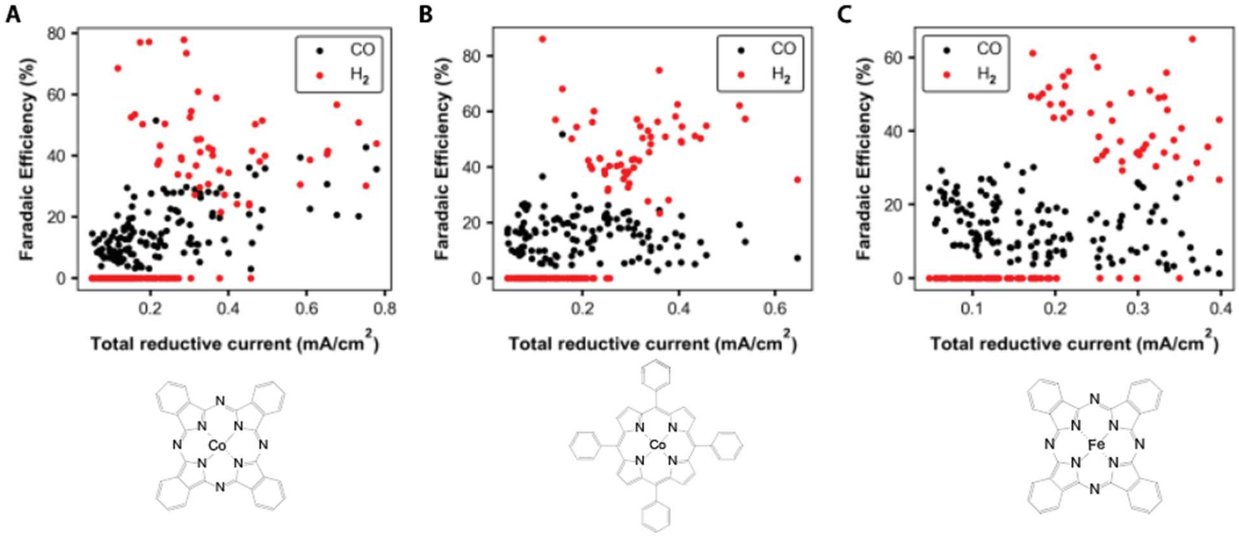

Figure S12. Faradaic efficiency towards both CO and H_2, aggregated across all runs based on total reductive current, for the three different catalysts. **A**: CoPc; **B**: CoTPP; **C**: FePc.

5. Additional discussion about mechanisms

5.1 Inventory of all models tested

Below we detail all models that were evaluated against the kinetic data. For each model, we provide a reaction schematic, the rate law equation, and values of the fitted parameters. Step-by-step derivations of rate laws for models presented in the main text are provided in SI Section 5.2. For the reaction schema, the following key can be used to understand the color coding and notation (Figure S13). Models that capture all key trends are boxed in green, and other models that missed one or more key experimental trends are boxed in orange.

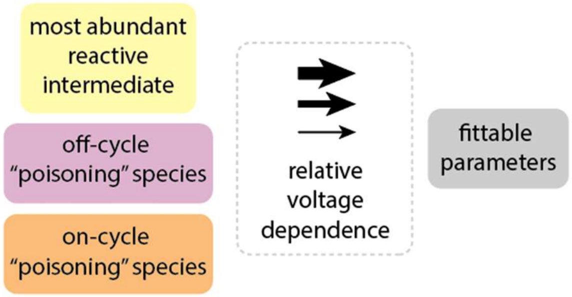

Figure S13. Key for the color coding and arrow sizes of the models presented in Figures S14 through S18.

5.1.1 Models for CoPc and CoTPP

The kinetic model shown in the main text is represented here as model A-1 (Figure S14, Figure 2G).

We also tried Model A-2, which could capture the bicarbonate order dependence via an "on-cycle" bicarbonate poisoning species (Figure S15). We note that this model is able to capture the bicarbonate dependence trends, including bicarbonate inhibition, without invoking an actual bicarbonate poisoning step. This is because at less reductive potentials, the top reaction pathway is sluggish, and bicarbonate directs reaction flux towards θ_{H+}, and away from the more productive bottom pathway, which leads to apparent bicarbonate inhibition. In the main text, we opted to present Model A-1 due to consistency with literature precedent.

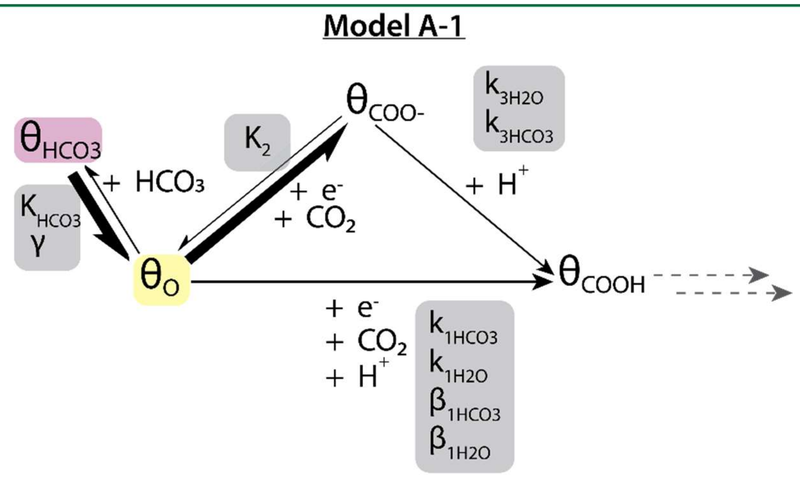

$$A = k_{1H2O}[CO_2]exp(\frac{-\beta_{1H2O}F\phi}{RT}) + k_{1HCO3}[HCO_3^-][CO_2]exp(\frac{-\beta_{1HCO3}F\phi}{RT})$$
$$B = k_{3H2O} + k_{3HCO3}[HCO_3^-]$$
$$C = K_2[CO_2]exp(\frac{-F\phi}{RT})$$
$$D = K_{HCO3}[HCO_3^-]exp(\frac{\gamma F\phi}{RT})$$

$$\mathbf{rate} = \frac{\mathbf{A+BC}}{\mathbf{1+C+D}}$$

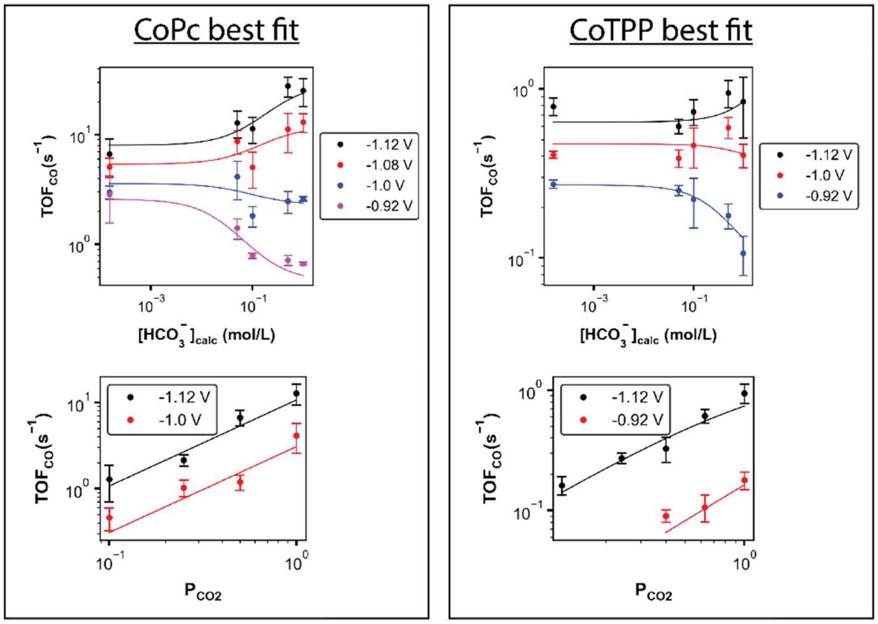

Parameter	$ln(k_{1H2O})$	$ln(k_{1HCO3})$	$ln(k_{3H2O})$	$ln(k_{3HCO3})$	$ln(K_2)$	$ln(K_{HCO3})$	β_{1H2O}	β_{1HCO3}	γ
CoPc	1.1	3.0	9.7	11.4	-15.5	3.2	0.10	0.19	0.32
CoTPP	-1.1	-1.7	-4.3	-1.2	-6.9	0.4	0.18	0.00	0.45

Figure S14. Model A-1. Full model that best describes the kinetic trends observed at CoPc and CoTPP. Mechanistic sketch, rate equation, graphs of model fit vs experimental data, and table of best-fit parameters are provided.

Model A-2

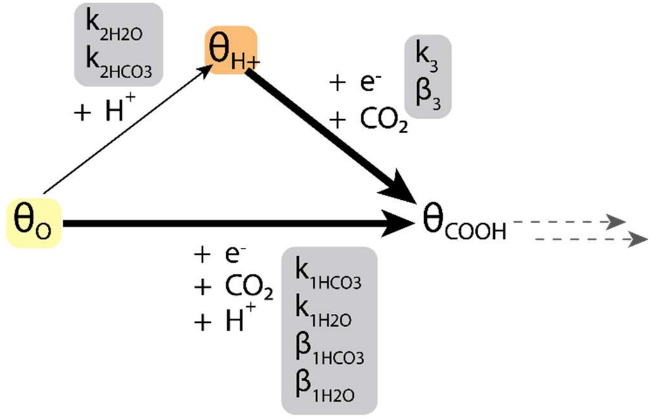

$$A = k_{1H2O}[CO_2]exp(\frac{-\beta_{1H2O}F\phi}{RT}) + k_{1HCO3}[HCO_3^-][CO_2]exp(\frac{-\beta_{1HCO3}F\phi}{RT})$$
$$B = k_{2H2O} + k_{2HCO3}[HCO_3^-]$$
$$C = k_3[CO_2]exp(\frac{-\beta_3 F\phi}{RT})$$

$$\mathbf{rate} = \frac{\mathbf{A+B}}{\mathbf{1+B/C}}$$

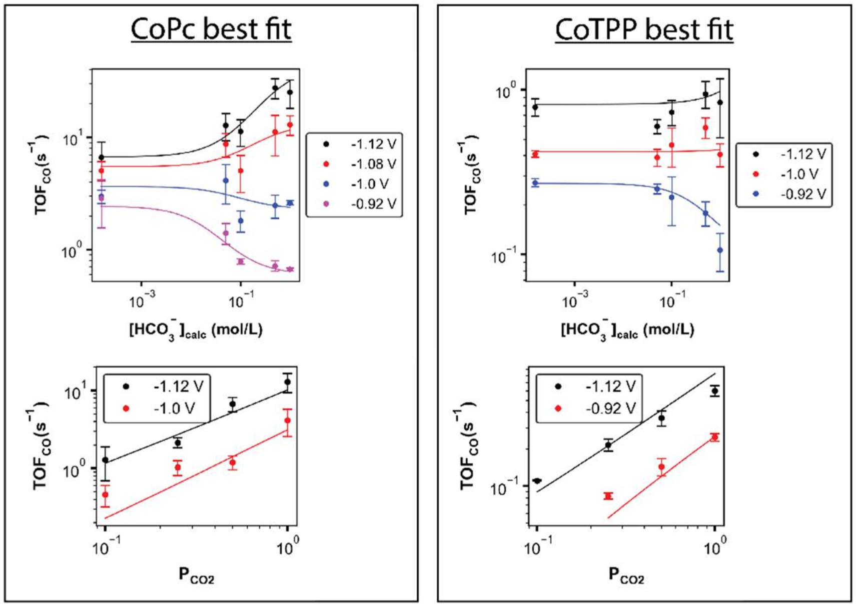

Parameter	$ln(k_{1H2O})$	$ln(k_{1HCO3})$	$ln(k_{2H2O})$	$ln(k_{2HCO3})$	$ln(k_3)$	β_{1H2O}	β_{1HCO3}	β_3
CoPc	1.0	-0.1	-20.0	3.3	-0.1	0.13	0.64	0.40
CoTPP	-1.1	-20.0	-19.5	-1.6	-1.8	0.14	0.26	0.51

Captures trends in bicarbonate poisoning
Cannot capture attenuation in CO_2 order dependence

Figure S15. Model A-2. Model is able to describe the bicarbonate order trends seen at CoPc and CoTPP. Mechanistic sketch, rate equation, graphs of model fit vs experimental data, and table of best-fit parameters are provided.

5.1.2 Explaining kinetic trends at FePc

We first attempted to explain the trends at FePc with Model B-1 (Figure S6), which invokes an intermediate later in the catalytic cycle, possibly some sort of adsorbed CO species, as the most abundant reactive intermediate (MARI). Although this model is able to capture the trends of how the bicarbonate and CO_2 dependences change with applied voltage, it is unable to capture the crossing behavior that occurs in the CO_2 order dependence.

Model B-1

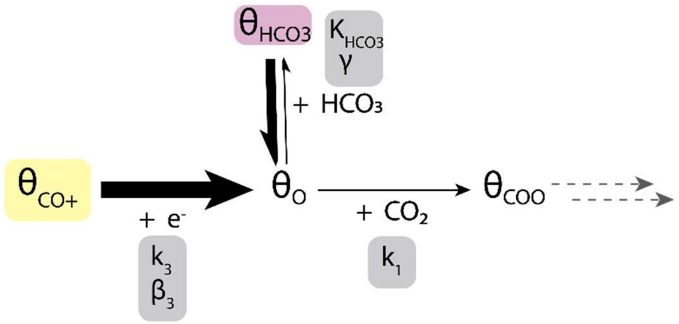

$$A = k_1[CO_2]$$
$$C = k_3 exp(\frac{-\beta_3 F\phi}{RT})$$
$$D = K_{HCO3}[HCO_3^-]exp(\frac{\gamma F\phi}{RT})$$

$$\mathbf{rate} = \frac{\mathbf{A}}{\mathbf{1+A/C+D}}$$

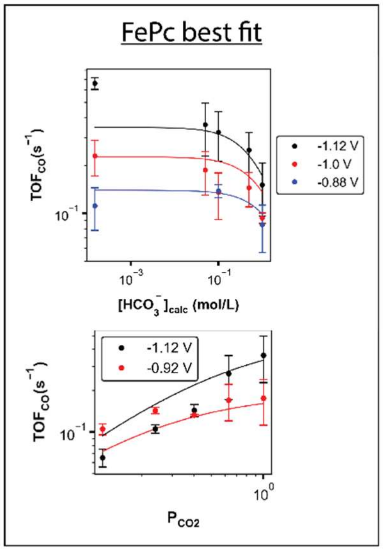

Parameter	ln(k1)	ln(k3)	ln(KHCO3)	β3	γ
FePc	0.1	-1.5	1.2	0.12	0.00

Does not capture cross in CO_2 order dependence

Figure S16. Model B-1. Model is only able to describe the direction of the bicarbonate and CO_2 trends seen at FePc. Crossing behavior in the CO_2 dependence remains unexplained. Mechanistic sketch, rate equation, graphs of model fit vs experimental data, and table of best-fit parameters are provided.

We then attempted to integrate the possibility of a late MARI, which was the essence of Model B-1, with the additional kinetic details of Model A-1 that were able to describe the CoPc and CoTPP data. This gave us Model B-2 (Figure S). However, this model was still unable to account for the crossing behavior in the CO_2 order dependence. Additionally, model fitting appeared to be under-constrained, where upon randomly varying the initial parameter guesses, we found many local solutions with qualitatively different kinetic behavior.

Model B-2

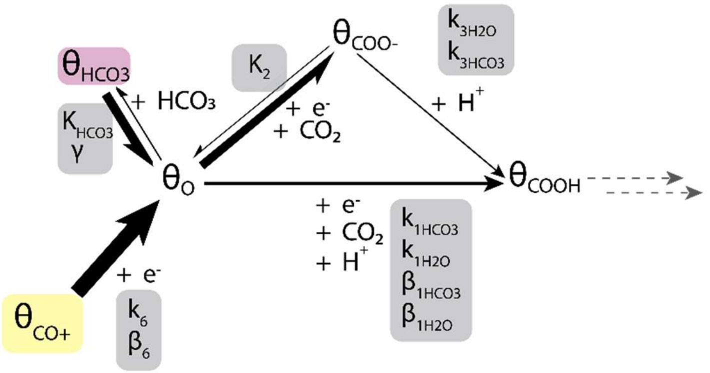

$$A = k_{1H2O}[CO_2]exp(\frac{-\beta_{1H2O}F\phi}{RT}) + k_{1HCO3}[HCO_3^-][CO_2]exp(\frac{-\beta_{1HCO3}F\phi}{RT})$$
$$B = k_{3H2O} + k_{3HCO3}[HCO_3^-]$$
$$C = K_2[CO_2]exp(\frac{-F\phi}{RT})$$
$$D = k_6 exp(\frac{-\beta_6 F\phi}{RT})$$
$$E = K_{HCO3}[HCO_3^-]exp(\frac{\gamma F\phi}{RT})$$

$$\mathbf{rate} = \frac{\mathbf{A+BC}}{\mathbf{1+C+A/D+E}}$$

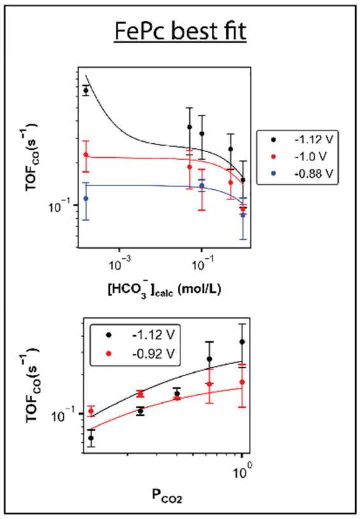

Parameter	$ln(k_{1H2O})$	$ln(k_{-1HCO3})$	$ln(k_{3H2O})$	$ln(k_{3HCO3})$	$ln(K_2)$	$ln(k_6)$	$ln(K_{HCO3})$	β_{1H2O}	β_{1HCO3}	β_6	γ
FePc	0.2	-20.0	-20.0	7.7	-6.6	-1.6	1.2	0.00	0.00	0.11	0.00

Does not capture cross in CO_2 order dependence
Fitting is underconditioned and many local solutions were found

Figure S17. Model B-2. Model is only able to describe the direction of the bicarbonate and CO_2 trends seen at FePc. Crossing behavior in the CO_2 dependence remains unexplained. Mechanistic sketch, rate equation, graphs of model fit vs experimental data, and table of best-fit parameters are provided.

Finally, we constructed Model B-3 (Figure S18, Figure 3D). We note that while the corresponding rate equation is able to recapitulate the experimentally observed kinetic trends, this model makes some very specific assertions. In particular, this model implies that CO desorption can occur both with (k_1) and without (k_3) an accompanying electron transfer event, that the resulting sites (θ_O vs θ_{O+}) have different affinities for bicarbonate and different reactivities with CO_2. Thus, the specific chemistry of this model may be taken with a grain of salt; its use is mainly in displaying the mechanistic features that could explain the complex experimental trends observed.

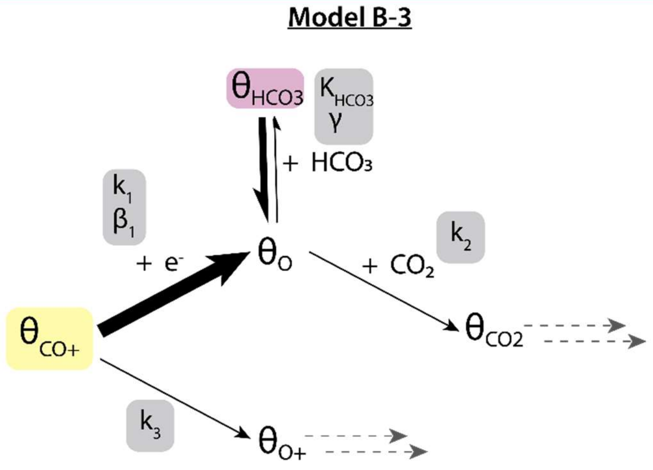

$$A = k_1 exp(\frac{-\beta_1 F\phi}{RT})$$
$$B = k_2[CO_2]$$
$$C = k_3$$
$$D = K_{HCO3}[HCO_3^-]exp(\frac{\gamma F\phi}{RT})$$
$$\textbf{rate} = \frac{\textbf{A+C}}{\textbf{1+A/B+AD/B}}$$

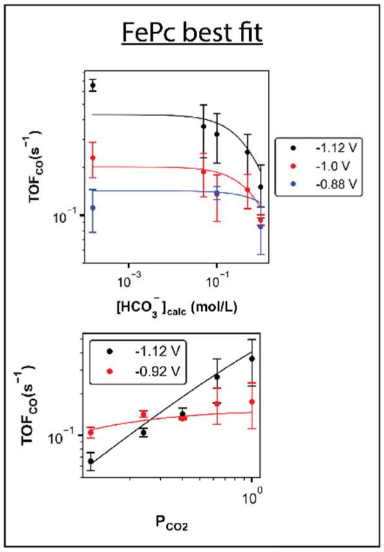

Parameter	ln(k1)	ln(k2)	ln(k3)	ln(KHCO3)	β1	γ
FePc	-3.4	-0.6	-2.0	2.0	0.57	0.23

Figure S18. Model B-3. A model that best describes the kinetic trends observed at FePc Mechanistic sketch, rate equation, graphs of model fit vs experimental data, and table of best-fit parameters are provided.

5.2 Derivation of rate laws for proposed mechanisms

5.2.1 Rate law for CoPc and CoTPP (Model A-1)

For clarity, the mechanism from main text Figure 2G (model A-1) is reproduced below with some additional reaction labels, where the name of each elementary step is labeled in red, and the associated parameters are highlighted in grey.

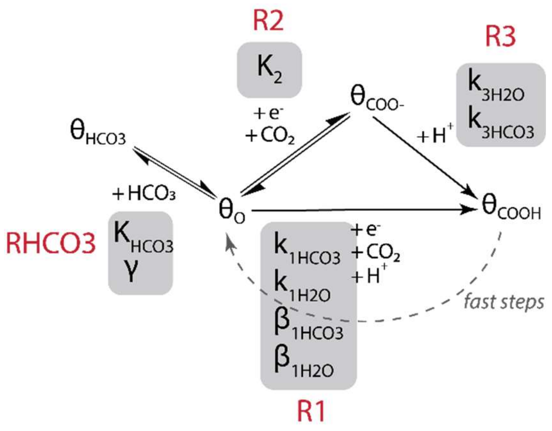

Figure S19. Annotated version of Figure 2G, where reaction paths and associated microscopic variables are explicitly labeled

The overall rate is given by the sum of rates of the top and bottom reaction pathways:

$$Rate = R1 + R3$$

Where based on the stoichiometries of the reactants, assumption of Bulter-Volmer electron transfer kinetics, and utilization of the defined kinetic parameters, the rates of elementary steps R1 and R3 are given below (the concentration of water is assumed to be constant, and thus is not explicitly written; it can be assumed to be baked in to the corresponding rate constants):

$$R1 = \left\{k_{1H2O}[CO_2]\, exp\left(\frac{-\beta_{1H2O}F\phi}{RT}\right) + k_{1HCO3}[HCO_3^-][CO_2]\, exp\left(\frac{-\beta_{1HCO3}F\phi}{RT}\right)\right\}\theta_O \equiv A\theta_O$$

$$R3 = \{k_{3H2O} + k_{3HCO3}[HCO_3^-]\}\theta_{COO-} \equiv B\theta_{COO-}$$

To now solve for θ_O and θ_{COO-}, which are the only unknowns in the above equation, we use the equilibrium conditions of R2 and RHCO3, as well as a site balance. For RHCO3, we are assuming an equilibrium condition that depends on the applied potential and the electrosorption valency (γ):

$$\theta_{COO} \quad = \left\{K_2[CO_2]\, exp\left(\frac{-F\phi}{RT}\right)\right\}\theta_O \equiv C\theta_O$$

$$\theta_{HCO} \quad = \left\{K_{HCO3}[HCO_3^-]\, exp\left(\frac{\gamma F\phi}{RT}\right)\right\} \equiv D\theta_O$$

$$\theta_O + \theta_{COO-} + \theta_{HC} \quad = 1$$

This leads to the solution that:

$$\theta_O = \frac{1}{1 + C + D}$$

$$\theta_{COO-} = \frac{C}{1 + C + D}$$

Plugging back in to the original rate equation, we get:

$$Rate = \frac{A + BC}{1 + C + D}$$

Where, as defined above:

$$A = k_{1H2O}[CO_2]\,exp\left(\frac{-\beta_{1H2O}F\phi}{RT}\right) + k_{1HC}\quad [HCO_3^-][CO_2]\,exp\left(\frac{-\beta_{1HCO3}F\phi}{RT}\right)$$

$$B = k_{3H2O} + k_{3HCO3}[HCO_3^-]$$

$$C = K_2[CO_2]\,exp\left(\frac{-F\phi}{RT}\right)$$

$$D = K_{HCO3}[HCO_3^-]\,exp\left(\frac{\gamma F\phi}{RT}\right)$$

5.2.2 Rate law derivation for FePc (Model B-3)

For clarity, the mechanism from main text Figure 3D (model B-3) is reproduced below with some additional reaction labels, where the name of each elementary step is labeled in red, and the associated parameters are highlighted in grey.

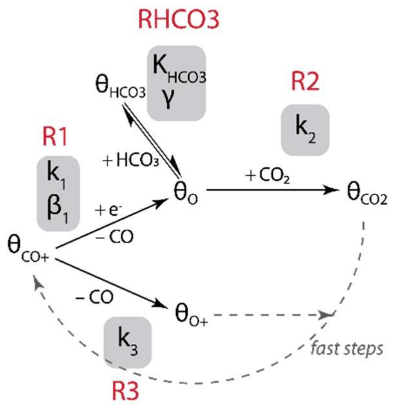

Figure S20. Annotated version of Figure 3D, where reaction paths and associated microscopic variables are explicitly labeled

The overall rate is given by the sum of the top and bottom reaction pathways. We note that the rate of the top pathway can be given by either R1 or R2, because we will later assume pseudo-steady-state, where R1 = R2. For convenience we will choose R1:

$$Rate = R1 + R3$$

Where based on the stoichiometries of the reactants, assumption of Bulter-Volmer electron transfer kinetics, and utilization of the defined kinetic parameters, the rates of elementary steps R1 and R3 are given as:

$$R1 = \left\{k_1\, exp\left(\frac{-\beta_1 F\phi}{RT}\right)\right\}\theta_{CO} \quad \equiv A\theta_{CO+}$$

$$R3 = \{k_3\}\theta_{CO+} \equiv C\theta_{CO}$$

To solve for θ_{CO+}, we will use a combination of a pseudo-steady-state approximation on θ_O (namely, that R1=R2), the equilibrium condition for bicarbonate poisoning, and a site balance. Again, for bicarbonate poisoning, we are assuming an equilibrium condition that depends on the applied potential and the electrosorption valency (γ):

$$\theta_O = \left\{\frac{A}{k_2[CO_2]}\right\}\theta_{CO+} \equiv \frac{A}{B}\theta_{CO+}$$

$$\theta_{HCO3} = \left\{K_{HCO3}[HCO_3^-]\, exp\left(\frac{\gamma F\phi}{RT}\right)\right\} \equiv D\theta_O$$

$$\theta_O + \theta_{CO+} + \theta_{HC} \quad = 1$$

Putting the above three together, we arrive at:

$$\theta_{CO+} = \frac{1}{1 + {}^{A}\!/_{B} + {}^{DA}\!/_{B}}$$

And substituting this back into the original rate equation gives:

$$Rate = \frac{A + C}{1 + {}^{A}\!/_{B} + {}^{DA}\!/_{B}}$$

Where, as defined above:

$$A = k_1\, exp\left(\frac{-\beta_1 F\phi}{RT}\right)$$

$$B = k_2[CO_2]$$

$$C = k_3$$

$$D = K_{HCO3}[HCO_3^-]\, exp\left(\frac{\gamma F\phi}{RT}\right)$$
