# Supplementary material for: Nonidealities in CO2 Electroreduction Mechanisms Revealed by Automation-Assisted Kinetic Analysis
Source: ACS Cent Sci. 2024 Jun 28;10(7):1348–56. doi: 10.1021/acscentsci.3c01295 (PMC11273456; doi:10.1021/acscentsci.3c01295)
Supplement: Supplementary file 2 — oc3c01295_si_002.pdf [file oc3c01295_si_002.pdf]

oc-2023-012958.R1

Name: Peer Review Information for "Non-idealities in CO₂ electroreduction mechanisms revealed by automation-assisted kinetic analysis"

First Round of Reviewer Comments

Reviewer: 1

Comments to the Author

In this manuscript, Zeng et al. performed cohesive electrokinetic analyses for CO2RR with an automated system that aims at improving the efficiency of the workflow for electrokinetic data collection. The cohesive kinetic analysis, unlike linearized kinetic technique such as Tafel plot or reaction order, provides complex mechanistic insights that are dictated by multiple parameters. Such analysis would require large number of electrochemical measurements, the efficiency of which is greatly enhanced by the applied automation reaction system. Despite being a proof-of-concept study, and the results of the electrochemical measurements are not perfect, I believe this work is worthy of publication in ACS Central Science. The automation-assisted electrokinetic system is a promising platform, assuming further improvement in the cell design and data robustness could be achieved, for enabling large volume of electrokinetic data collection that is not only beneficial to the study of CO2RR, but other electrochemical reactions. Therefore, this work is of interest to a broad electrochemical research community. I only have some minor comments for the authors:

1. I agree that the automated reaction system is a good approach to enhance the efficiency of the kinetic, mechanistic studies, which typically demands large volume of and tedious data collection. One concern for the automated reaction is that, prior to each electrolysis, a single cell pan is purged with CO2 gas for ~ 5 min before potential is applied. Also, the electrolysis in each “cell” was conducted in a fairly short time. I wonder if these are sufficient time lengths to ensure a “steady state” CO2RR reaction. This is related to the credibility and reproducibility of the electrolysis. The authors can provide some experimental data to prove if the adopted protocol generates stable and reproducible results.

2. The mechanistic demonstrations in Figure 2 and 3 are confusing and very difficult to follow. The nomenclatures of terms are poorly annotated in the main text and should be improved. In the SI, the authors provided kinetic models that “best” fit the experimental trend. The process makes sense to me, but considering the broad readership of ACS Central Science, I think the authors may expand the kinetic derivation with clear statement on the assumptions, step-by-step derivation of each rate law, so that the results could be more accessible to most readers.

Reviewer: 2

Comments to the Author

This manuscript details the development and application of a robotic system for analyzing the kinetics of the CO2 electroreduction reaction (CO2RR). The robotic system enables the sequential and automatic collection of electrochemical rate data from up to ten cells. The analysis reveals non-ideal kinetic features such as non-linearity and voltage dependence in the apparent bicarbonate orders, facilitated by the robotic system. To fit the experimental data, a series of kinetic models are proposed. The development and employment of automated reaction systems are highly desirable, however, as a novel approach, its reliability needs to be verified. The authors showed the general correlation between reported rates and those determined with the automated system, however, the absolute rates of the latter were about one order of magnitude lower (Fig. S8). This origin of this stark difference needs to be identified. Based on the pictures and schematics of the setup in the main text and SI, there is no forced convection during the CO2RR. This could induce severe mass transport limitation of CO2, and causing the lower measured rates. Since kinetic analysis was applied on the measured rates, an implicit assumption was all rates were kinetically controlled. This point must be verified experimentally for this work to be publishable.

Other comments & questions:

1. In the cell design of the robotic system, the potential interference of the crossover of the product of the counter electrode, e.g., oxygen, needs to be excluded.

2. Figures 2d-g and Figure 3d,e illustrate the proposed model for data fitting, but the notations are confusing. It is suggested to label every node with specified species (presented in supporting information) to enhance readability and provide more chemical information.

3. What is the major difficulty in obtaining a model with a significant F-test value? The models and anlysis methods presented in this manuscript are similar to the previous work (ACS Catal. 2020, 10 (7), 4326–4336.) , where F-test results were provided.

4. If the difficulty mentioned in the third comment arises from a lack of data, rate data corresponding to concentrations in the range from 10-3 to 10-2 should be replenished. A key advantage of an automated reaction system is the ease of obtaining large amounts of reproducible data. However, reactivity data in this work do not appear to be more abundant than existing work without the automated system, and the experimental errors are also not smaller. Thus, kinetically rigorous data at more concentrations and narrower spread should be provided to increase the reliability of the kinetic analysis.

5. Operating workflow and protocol are important for a robotic system. A flow chart should be provided to demonstrate how to make the full use of the 10 cells in the system.

Author's Response to Peer Review Comments:

Karthish Manthiram
Professor of Chemistry and Chemical Engineering manthiramlab.com | karthish@caltech.edu

April 21, 2024

Senior Editor
ACS Central Science

Re: Non-idealities in CO2 electroreduction mechanisms revealed by automation-assisted kinetic analysis

Dear Editor,

Thank you for facilitating such a thoughtful review of our manuscript. My colleagues and I have taken into consideration the reviewers' comments to further improve the work and are resubmitting a revised manuscript.

Encouragingly, the reviewers were excited about our work, finding it to be "of interest to a broad electrochemical research community". The reviewers agreed that "the development and employment of automated reaction systems are highly desirable" and found our study to be "a promising platform", that despite being proof-of-concept, paves the way for the development of automated systems that would "enable[e] large volume[s] of electrokinetic data collection that is not only beneficial to the study of CO2RR, but other electrochemical reactions".

The reviewers' criticisms largely focused on further demonstrating robustness of the kinetic data and improving the clarity of the mechanistic discussions. To address this feedback, we have included new experimental flow rate dependence data, additional analyses of rate stability, and revised mechanistic figures. Additionally, we have made significant edits to mechanistic explanations and derivations in both the main text and SI. Key revisions in the manuscript and SI are highlighted in yellow, and we are attaching a point-by-point response to the reviewers' comments.

Reviewer 1 suggested we provide further evidence that we were measuring steady-state rates with our protocol. To address this, we added a new stability analysis of our rate data. Additionally, Reviewer 1 suggested improving the clarity of the mechanistic discussion and adding derivations for the presented rate laws. To address this, we made significant edits, including two revised main text figures, two revised main text sections, and one new SI section.

Reviewer 2's comments also largely focused on validating the robustness of the experimental approach. The reviewer expressed concerns about mass transport, anode-cathode crosstalk, and the lack of a quantitative model fit for the data. For the first concern, we performed a flow rate dependence, which is the canonical experimental test for external mass transport limitations. Unfortunately, we were not able to implement any changes to address the second and third concern, because the associated experimental burden is outside the scope of this initial

demonstration. In lieu of this, we have provided explanations in the rebuttal for why, despite these shortcomings, our work still provides a useful and foundational demonstration of an automated electrochemical data collection platform. Finally, Reviewer 2 also suggested improving the clarity of the mechanistic discussion and adding more details about operational workflow; we addressed these with significant edits, which included the revisions that we made to address Reviewer 1's suggestion, as well as one more revised main text figure and one more new SI section.

We hope that we have sufficiently addressed the concerns of the reviewers both in the manuscript and in our responses. If you have any concerns regarding our reviewer responses or manuscript, please do not hesitate to let us know.

Sincerely,

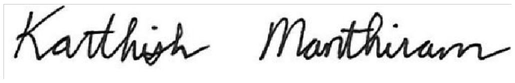

Karthish Manthiram

Professor of Chemistry and Chemical Engineering California

Institute of Technology

oc-2023-012958.R2

Name: Peer Review Information for "Non-idealities in CO₂ electroreduction mechanisms revealed by automation-assisted kinetic analysis"

Second Round of Reviewer Comments

Reviewer: 1

Comments to the Author

My comments have been properly addressed by the authors, I therefore recommend publication of this manuscript as it is.

Reviewer: 2

Comments to the Author

The authors have adequately addressed my comments, and the manuscript is recommended to be published as it is.

Author's Response to Peer Review Comments:

Dear Editor,

We are thrilled for the opportunity to publish our work in ACS Central Science! Please find our revised manuscript attached. To address the requested formatting needs, we have added a synopsis in the manuscript file after the TOC graphic.

Additionally, we added one statement in the acknowledgements to acknowledge that the manuscript is adapted from a PhD thesis.

Please let me know if we can provide any additional information.

Sincerely,

Karthish Manthiram
